# Quantum chemical calculation of electron ionization mass spectra for general organic and inorganic molecules†
†Electronic supplementary information (ESI) available: GFN-xTB calculated potential energy surfaces for example coordinates. Additional calculated mass spectra. Computational timing statistics. See DOI: 10.1039/c7sc00601b



**DOI:** 10.1039/c7sc00601b

**Published:** 2017-05-05

**Authors:** Vilhjálmur Ásgeirsson, Christoph A. Bauer, Stefan Grimme

**Affiliations:** a Mulliken Center for Theoretical Chemistry , Institute of Physical and Theoretical Chemistry , University of Bonn , Beringstr. 4 , 53115 Bonn , Germany . Email: grimme@thch.uni-bonn.de ; Tel: +49 228 73 2351; b Faculty of Physical Sciences and Science Institute , University of Iceland , 107 Reykjavík , Iceland

## Abstract

We introduce a fully stand-alone version of the Quantum Chemistry Electron Ionization Mass Spectra (QCEIMS) program [S. Grimme, *Angew. Chem. Int. Ed.*, 2013, **52**, 6306] allowing efficient simulations for molecules composed of elements with atomic numbers up to *Z* = 86. The recently developed extended tight-binding semi-empirical method GFN-xTB has been combined with QCEIMS, thereby eliminating dependencies on third-party electronic structure software. Furthermore, for reasonable calculations of ionization potentials, as required by the method, a second tight-binding variant, IPEA-xTB, is introduced here. This novel combination of methods allows the automatic, fast and reasonably accurate computation of electron ionization mass spectra for structurally different molecules across the periodic table. In order to validate and inspect the transferability of the method, we perform large-scale simulations for some representative organic, organometallic, and main-group inorganic systems. Theoretical spectra for 23 molecules are compared directly to experimental data taken from standard databases. For the first time, realistic quantum chemistry based EI-MS for organometallic systems like ferrocene or copper(ii)acetylacetonate are presented. Compared to previously used semiempirical methods, GFN-xTB is faster, more robust, and yields overall higher quality spectra. The partially analysed theoretical reaction and fragmentation mechanisms are chemically reasonable and reveal in unprecedented detail the extreme complexity of high energy gas phase ion chemistry including complicated rearrangement reactions prior to dissociation.

## Abstract 2

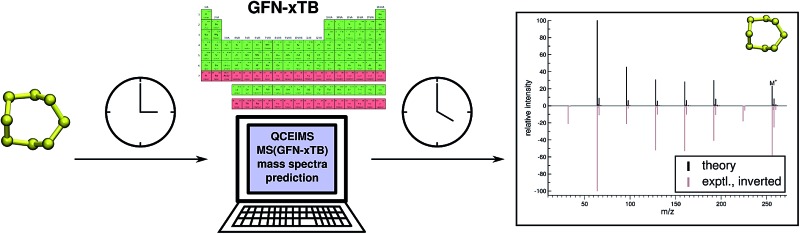
The implementation of a novel tight-binding Hamiltonian within the QCEIMS program allows the first-principles based computation of EI mass spectra within a few hours for systems containing elements up to *Z* = 86.

## 1 Introduction

The application of *ab initio* molecular dynamics (AIMD)^1^ has become increasingly popular in recent years with the growing routinely available computational resources and the advent of efficient electronic structure methods and algorithms. In AIMD the chemical dynamics of a system is simulated directly by classically propagating the nuclear degrees of freedom, where the atomic forces along a potential energy surface (PES) are computed on the fly by a given quantum chemistry (QC) method. Recently, AIMD has been increasingly employed in relation to mass spectrometry to aid in the interpretation of, or even predict, experimental results *e.g.*, in electron ionization (EI) mass spectrometry,^2,3^ collision induced dissociation (CID),^4–6^ surface induced dissociation,^7–9^ and dissociative electron attachment (DEA).^10–12^ AIMD simulations provide a promising alternative to the well-established statistical theories (*e.g.*, Eyring's quasi-equilibrium theory,^13^ and Rice–Ramsperger–Kassel–Marcus (RRKM) theory^14–17^). The problem of defining a set of decomposition channels *a priori* and locating the respective stationary points (minima and saddle points) on the potential energy surface (PES) is entirely avoided. As the number of viable decomposition channels grows rapidly with increasing molecular size, it can become very tedious and in some cases even biased to use a statistical treatment for large molecular systems. However, if care is taken, such treatment can be very useful and yield valuable insights to mechanistic studies, by comparing the relative microcanonical rate constants for different unimolecular decomposition pathways. Therefore, RRKM theory has been widely applied in the context of mass spectrometry.^18^

AIMD simulations are able to explore automatically the energetically available regions of phase space and yield decomposition channels in an unbiased fashion. However, AIMD simulations are computationally expensive. Large-scale simulations beyond the picosecond time scale using density functional theory (DFT), or highly accurate wave function methods become computationally prohibitive. On these terms, fast and numerically robust semi-empirical schemes^19^ provide a cost-efficient alternative.

Semi-empirical electronic structure methods are constructed by applying various approximations to Hartree–Fock (HF), yielding methods like the Parametric Models (PMx)^20,21^ and the Orthogonalization-corrected Models (OMx).^22^ More recently such approximations have been applied to DFT, in particular to the exchange–correlation (XC) functional PBE,^23^ known as the DFTBx series.^24,25^ These methods retain the fundamental limitation of the respective HF/DFT parent method, introduce further approximations to electronic integrals (*e.g.*, the neglect of three and four center integrals and the use of two center integral approximations) and employ minimal valence basis sets. Furthermore, the parametrization of a particular semi-empirical method often yields a poor description of molecular systems which differ from the training set and for properties that have not been included. The aforementioned approximations lead to an increase in computational efficiency by up to three orders of magnitude compared to HF/DFT. The price to pay is lowered accuracy and a poor description of certain chemical features. The considerable efforts devoted to develop corrections to these problems are summarized in ref. 19. The high computational throughput of these methods render them valuable tools in large-scale quantum chemical calculations, *e.g.*, for biomolecular applications (>1000 atoms)^19,26^ and long time-scale AIMD.

**Fig. 1:**
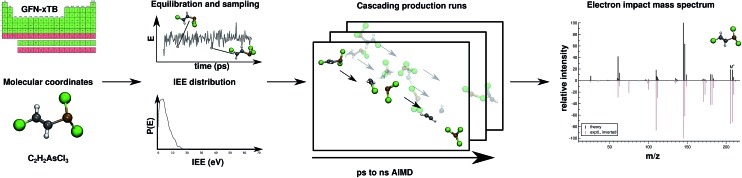
Overview of the QCEIMS protocol. The number of production runs is chosen such that the simulation results are statistically converged.

Regarding EI mass spectrometry, an original, exhaustive and widely applicable AIMD protocol has been devised and published, it is referred to as the Quantum Chemistry Electron Ionization Mass Spectra (QCEIMS) program.^2^ It is an automated, easy-to-use, dynamical procedure which combines AIMD with stochastic and statistical elements in order to predict reasonably accurate EI mass spectra (EI-MS) (see Fig. 1), without any preconceived notion of decomposition pathways. However, an almost non-empirical unbiased brute-force approach can not compete for fundamental reasons with the accuracy of database driven, knowledge based EI-MS predictors,^27–30^ which should be kept in mind when judging the theoretical spectra.

The program is coupled to various third-party electronic structure software (*e.g.*, MOPAC,^31^ ORCA,^32,33^ TURBOMOLE^34^), allowing the atomic forces required by the QCEIMS internal molecular dynamics procedure to be calculated with various semi-empirical methods (*e.g.*, DFTB3, OM2, PM3 and PM6) and standard DFT functionals. One of the main strengths of QCEIMS is that a simulated EI-MS result can be directly compared to the experiment. In addition the simulations provide valuable mechanistic insights into the dissociation dynamics, where bond ruptures and even complex molecular rearrangements prior to decomposition automatically occur during the simulations. All simulation trajectories are stored and can thus easily be inspected or post-processed. Therefore, the procedure is able to aid the user in mechanism-to-fragment-to-peak assignment. The decomposition pathways can also signify which channels are of high importance and can therefore be used in tandem with statistical theories, refined by higher level QC methods. In fact, DFT calculations have been used in the literature to study mass spectral fragmentation pathways, although the pathways were largely found because of prior experimental knowledge.^35–39^

A number of studies were conducted using the QCEIMS protocol on organic drug molecules^40^ and the nucleobases^41,42^ using different semi-empirical QC methods. In the most recent study, QCEIMS was extended to successfully predict the unimolecular decomposition pathways of four negatively charged nitrile compounds upon low energy electron attachment.^12^

In this study we have implemented two new semi-empirical methods, the GFN-xTB^43^ and IPEA-xTB, in the QCEIMS program allowing spectral simulations for basically any reasonable molecule from the periodic table in a matter of minutes to a few hours of computation time, depending on the simulation conditions and number of available cores. The quantum chemical methods are tight-binding (TB) electronic structure schemes, where the former method was independently developed to accurately describe molecular geometries, atomic forces and non-covalent interactions of large molecules. The latter version of the same TB Hamiltonian was developed to accurately compute ionization potentials (IPs) and electron affinities (EAs), for QCEIMS as well as electrochemical applications (which are published separately). The GFN-xTB has been reported to be more computationally efficient, robust, and globally accurate than other similar semi-empirical methods, for the listed target properties.^43^ Moreover, GFN-xTB has parameters available for elements with atomic numbers up to *Z* = 86, making the approach applicable to a large range of molecular systems. Here we present the first, fully standalone version of QCEIMS, where GFN-xTB is used for all energy and gradient computations. Note, that GFN-xTB which provides the PES for all reactions investigated in this work, was not modified specifically for the purpose studied here. Most IP evaluations, which are needed to compute the charge distribution on fragments, are conducted with the IPEA-xTB variant. This second parametrization is needed because at the TB level one can not simultaneously describe good PES and IP/EAs. Our new developments eliminate the necessity to employ third-party electronic structure software in QCEIMS and a fully stand-alone code is presented here for the first time. However, the option to use such software (*e.g.*, for DFT refinements) remains available. In the next section, a description is given for both of the TB variants and the QCEIMS protocol.

The purpose of this work is to assess the quality of simulated EI-MS produced by the combination of GFN-xTB/IPEA and QCEIMS along with its transferability to a diverse set of molecules. The basic QCEIMS scheme is not modified. Furthermore, the robustness and computational efficiency are investigated. For this purpose, we construct a molecular test set of 23 diverse molecules, composed of 24 different chemical elements. There are two criteria for the selection of the molecules. The first objective is to include as many elements as possible, in order to validate that the approach can predict accurate EI-MS for molecules composed of elements across the periodic table. The second objective is to compare the simulated spectra directly to the experimental spectra. Therefore, the molecules have to be well-known with well validated experimental EI-MS. All systems are obtained from the NIST^44^ and SDBS^45^ databases. Furthermore, the molecules should vary in structure, size, and chemical functionality. The chosen molecules are divided into three groups, organic, organometallic and main-group inorganic molecules. QCEIMS results for the later two groups are presented here for the first time.

**Fig. 2:**
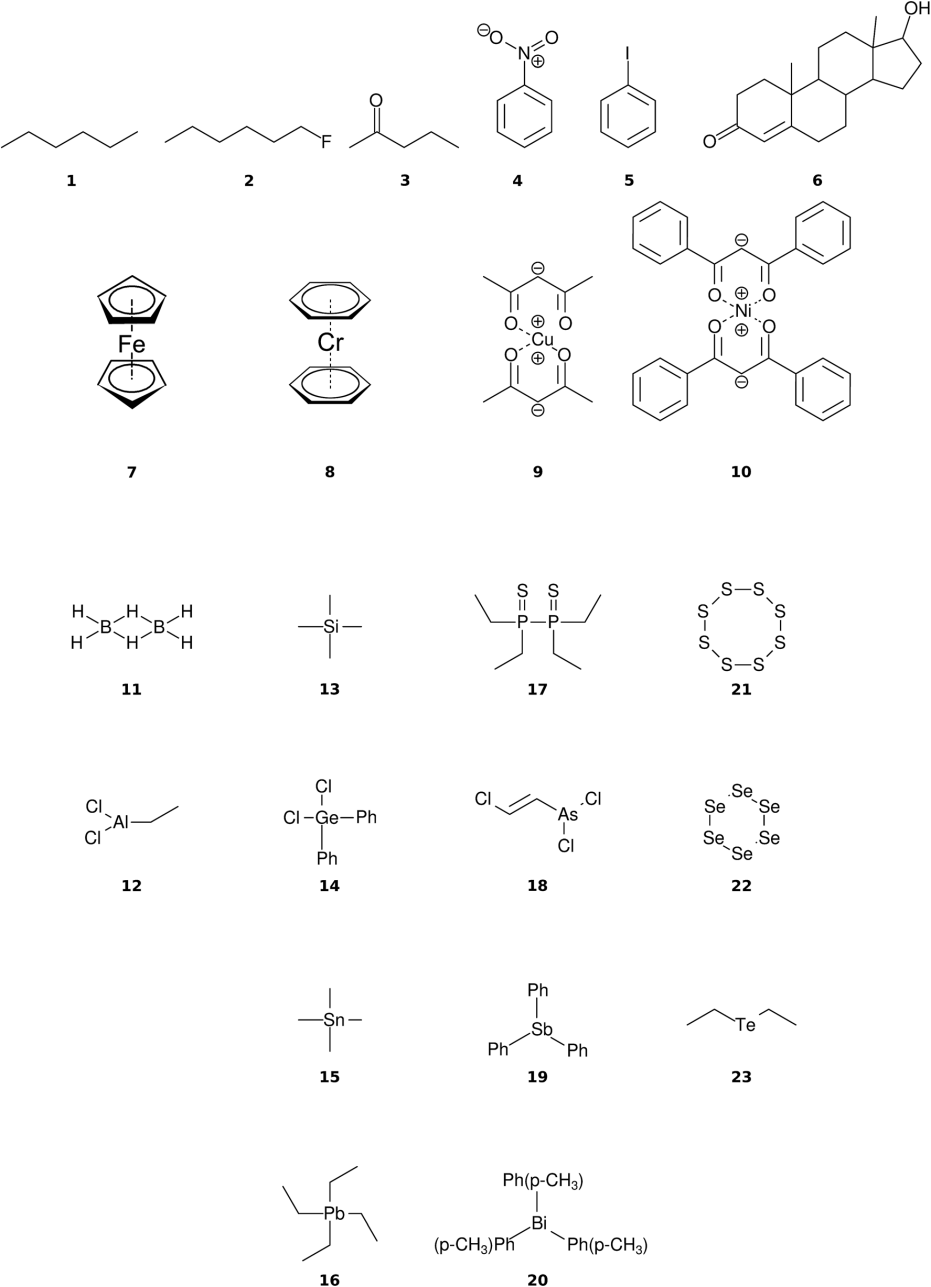
Chart of the molecular test set (**1–23**). The first two rows represent organic and organometallic molecules, respectively. The last four rows represent main group inorganic molecules, arranged by columns in analogy to the periodic table (groups 13–16). Phenyl groups are denoted by Ph.

The organic molecular group includes hexane (C_6_H_14_) (**1**), 1-flouro hexane (C_6_H_13_F) (**2**), 2-pentanone (C_5_H_10_O) (**3**), nitrobenzene (C_6_H_5_NO_2_) (**4**), iodobenzene (C_6_H_5_I) (**5**) and testosterone (C_19_H_28_O_2_) (**6**). The organometallic group includes ferrocene (C_10_H_10_Fe) (**7**), bis-benzene chromium (C_12_H_12_Cr) (**8**), copper(ii)acetylacetonate (C_10_H_14_O_4_Cu) (**9**), nickel(ii)bis(diphenyl-acetylacetonate) (C_30_H_22_O_4_Ni) (**10**). The main group inorganic molecules include diborane (B_2_C_6_) (**11**), dichloro-ethylaluminium (C_2_H_5_Cl_2_Al) (**12**), tetramethylsilane (C_4_H_12_Si) (**13**), dichloro-diphenylgermanium (C_12_H_10_Cl_2_Ge) (**14**), tetramethylstannane (C_4_H_12_Sn) (**15**), tetraethyllead (C_8_H_20_Pb) (**16**), tetraethyl-diphosphane-disulfide (**17**), lewisite (C_2_H_2_Cl_3_As) (**18**), triphenylstibine (C_18_H_15_Sb) (**19**), tris(*para*-tolyl)bismuthine (C_21_H_21_Bi) (**20**), octasulfur (S_8_) (**21**), selenium hexamer (Se_6_) (**22**) and diethyltelluride (C_4_H_10_Te) (**23**). In the inorganic molecular group, we have omitted molecules composed of elements for which EI-MS is not easily obtained *i.e.*, gallium, indium, thallium and polonium. Schematic representation of the molecules is given in Fig. 2, arranged in the order of the three given groups, organic (**1–6**), organometallic (**7–10**) and main group inorganic molecules (**11–23**). Furthermore, the inorganic molecules are arranged by columns in analogy to groups 13–16 of the periodic table. We believe that the chosen molecular set will attest to the wide applicability and accuracy of the novel approach presented here. We aim to encourage the community to use QCEIMS in tandem to traditional experimental mass spectrometry. The QCEIMS (3.62) program is available upon request^46^ and should be suitable for any Linux distribution.

The paper is organized as follows: in Section 2, a general description of QCEIMS is given accompanied with a brief description of the underlying GFN-xTB and IPEA-xTB methods. Moreover, the computational expenses and robustness of the methods are discussed. In Section 3, we report the simulated EI-MS for the above molecular test sets and compare the results directly to the respective experimental data. We discuss each molecule individually and address interesting decomposition pathways with an emphasis on molecular rearrangements. In Section 4, concluding remarks are given.

## 2 Methodology

### 2.1 QCEIMS

The QCEIMS procedure is executed for each molecule, in three steps: (i) equilibration and sampling of (neutral) conformers, (ii) calculating the molecular orbital spectrum and (iii) production runs. A somewhat concise description of the three steps is given in the following. For a more involved discussion of QCEIMS, the reader is referred to ref. 2. The first and last steps of the procedure involve MD, where the neutral molecule or its positive ion, respectively, is propagated in time by numerically integrating Newton's equations of motion using the leap-frog algorithm. The time step is 0.5 fs. The atomic forces needed to integrate the equations of motion are calculated on the fly using GFN-xTB, which has been implemented in the QCEIMS program. The combination of QCEIMS and GFN-xTB is referred to as MS(GFN-xTB) if IPEA-xTB is used for the IP calculation (and MS(GFN-xTB/DFT) if DFT is used instead for the IP calculations) in the following discussion.

#### 2.1.1 Equilibration and sampling (i)

The neutral molecule of interest is equilibrated over a period of 12.5 ps in the canonical ensemble (*NVT*), with a constant temperature of 500 K. The equilibration is followed by a conformer sampling in the micro-canonical ensemble (*NVE*), where 1000 snapshots (geometry and nuclear velocities) are randomly selected and saved along a 25.0 ps trajectory. For consistency, the same simulation time (or trajectory length) is used for all molecules. The time is chosen such that a statistically uncorrelated sample of conformers is ensured, even for the largest molecules in the test set *e.g.*, **6** and **10**. Very flexible systems, which are not considered here, will require longer ground state trajectories.

#### 2.1.2 Molecular orbital spectrum (ii)

A single-point calculation with MS(GFN-xTB) is performed to determine the molecular orbital (MO) spectrum, followed by an MO resolved Mulliken population analysis.^47^ This calculation is required, in order to estimate necessary ion state related quantities for the production run simulations, *i.e.*, the internal excess energy (IEE), internal conversion (IC) time, and MO-population derived nuclear velocity scaling factors.

The internal excess energy (IEE) represents the energy imparted on the molecule by the colliding electron and is distributed among the vibrational modes of the parent ion (also referred to as the molecular ion), by scaling the nuclear velocities (heating). The value of IEE for each production run is chosen in a stochastic manner, where it is assumed to be a Poisson-type variant,1
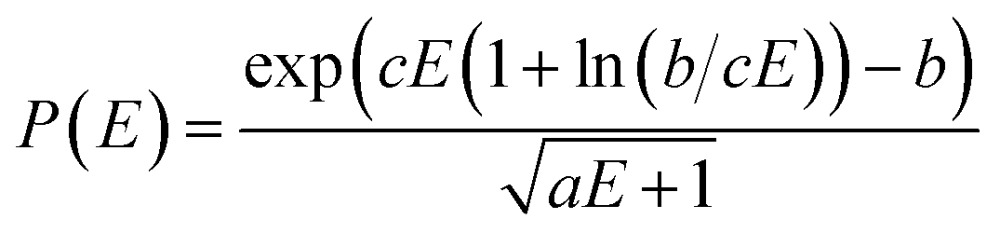

*P*(*E*) is the probability to have an IEE equal to *E*. The parameters *a*, *b* and *c* are given as ≈0.2 eV, 1.0 and 
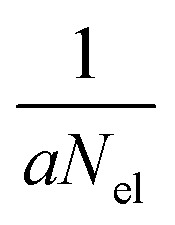
, respectively. The maximum value of IEE is equal to *E*_impact_ – *ε*_HOMO_, where *E*_impact_ is an input parameter and represents the kinetic energy of the free electron, before impact. It is set to 70 eV in analogy to standard EI experiments. The IEE distribution is set to have its mode at 0.6 eV per atom.

The internal conversion (IC) time is an interval over which the ion is heated. After the IC process, the IEE is entirely converted into nuclear kinetic energy. The IC time is calculated from the energy gap-law^48^ and is dependent on the differences in MO energies. It is given by,2
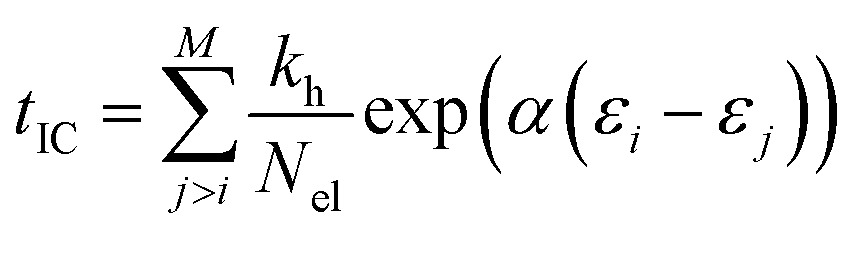
where *α* = 0.5 eV^–1^ and *k*_h_ = 2 ps. *M* is the ordinal number of the HOMO and *ε*_*i*_ is the orbital energy of the *i*-th orbital. For molecules consisting of less than 35 atoms, MO-based velocity scaling factors are used. The scaling factor of a particular nucleus is proportional to the Mulliken population of that nucleus in the ionized MO. The idea is that ionization of localized MOs will yield localized structural distortions and therefore induce decomposition in the spatial vicinity of the MO. For larger molecules, the velocity scaling factors have been observed to yield some artifacts^40^ (probably because the initial ionic states have more delocalised continuum character in larger systems) and are therefore set to unity.

#### 2.1.3 Production runs (iii)

The randomly sampled conformers are instantaneously (valence) ionized and the coordinates and nuclear velocities are used as initial conditions for the propagation of the molecular parent ion in 1000 individual production runs. The production runs are performed in an embarrassingly parallel manner. The large number of runs, for each molecule, is to ensure that the resulting EI-MS are statistically converged with respect to the observed fragments. Furthermore, the maximum simulation time of an individual production run is chosen to be 10 ps (compared to a default value of 5 ps, used previously), to reduce the number of cases where the parent ion would otherwise not decompose, because of too short simulation time. The effect of this maximum simulation time is investigated in more detail for the two cases **2** and **13** as shown in the ESI.† Note, that the overall simulation time in one run for a given parent molecular ion conformation can individually be much longer than the above maximum MD time of 10 ps because of the cascading technique used (see below).

In the beginning of each production run, the ion is heated by scaling the nuclear velocities, as described in the last subsection. The heating phase is usually conducted within the first 0.2–3.0 ps (IC time) of the simulation. The conceptual idea of the model is that after the EI of the molecule, an electronically excited ion will form, which relaxes to a vibrationally excited level of the electronic ground state (hot ion) through IC, followed by intramolecular vibrational redistribution (IVR), *i.e.*, the excess energy imparted on the molecule from the colliding electron is transferred to the vibrational modes of the ion. Further propagation can then result in decomposition of the parent ion to favorable (radical) neutral and charged moieties.

If fragmentation occurs the algorithm will evaluate the vertical IP of each product by a Δself-consistent field (SCF) or Δself-consistent charge (SCC) calculation (see below), using IPEA-xTB, which is a differently parametrized TB variant of xTB implemented in the QCEIMS and solely employed for calculations of IPs. For molecules with a more difficult electronic structure *e.g.*, transition metal complexes, it can become vital to use more accurate QC methods for the IP evaluations in order to obtain more accurate peak intensities. Therefore, we use PBE0/def2-SV(P)^49^ to evaluate the IPs for the organometallic molecules. All DFT calculations are performed using the ORCA 3.0.3 suite of programs. Moreover, a new feature is introduced in the QCEIMS protocol: when a fragment contains a 3d transition metal, the algorithm will begin by automatically finding the ground state multiplicity for both the ion and the neutral molecule, followed by the ΔSCF evaluation of the neutral and ion ground state. This new feature introduces few additional single point energy calculations but is found to improve the IPs. The statistical weight of each product is then given by3
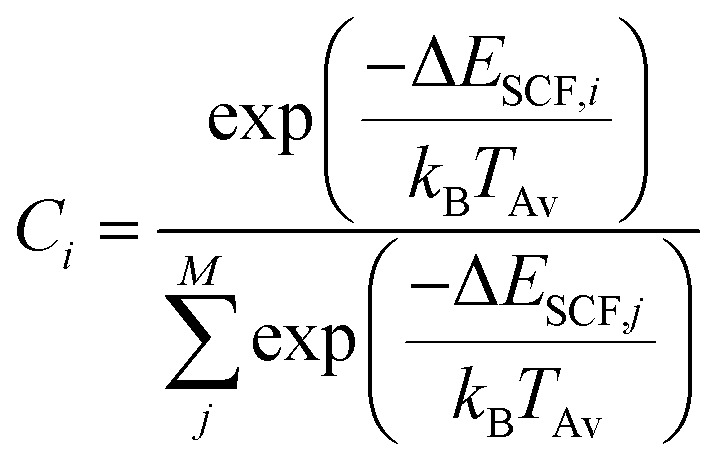
where *M* is the number of fragments, *T*_Av_ is the average fragment temperature and *k*_B_ is the Boltzmann constant. The product of the statistical weight *C*_*i*_ and the total molecular charge yields the statistical charge of fragment *i*.

The fragment with the highest statistical charge is selected and propagated further in a so-called cascade, while the other fragments (with lower statistical charges) are counted and stored. In the cascade, the selected positively charged secondary fragment can decompose further. If this secondary fragment decomposes, the IPs of the newly formed tertiary fragments are calculated and the statistical charges are determined. Again, the tertiary fragment with the highest statistical charge is selected and a new cascade initiated. In each cascading run, the statistical weights are multiplied by the dominant statistical weight of the preceding run. In other words, the sum of the statistical charges of all order fragments, in a single production run, including all cascades, is equal to the total molecular charge (*i.e.*, 1). Therefore, the sum of the statistical charges for a specific fragment over the ensemble of production runs will yield the total relative intensity of the particular fragment, allowing the algorithm to predict EI-MS for an arbitrary molecule, as long as the PES and IP computations are reasonable. The natural isotope ratios are introduced in a post-simulation treatment. Furthermore, specific isotope labeling can easily be introduced by performing the simulations with altered nuclear masses. However, this option is not considered herein. The quality of the resulting, fully theoretical, basically first-principle, MS is determined by a number of factors, in particular the underlying QC method. It has been observed that the level of QC accuracy is reflected in the quality of the simulated spectrum.^2,41^ Moreover, the number of production runs and the maximum simulation time accessible can affect the resulting spectra. More subtle effects which are harder to resolve are *e.g.*, the neglect of non-adiabatic effects (*i.e.*, where the charge is not assigned to the fragment with the lowest IP) and the nature of the IEE distribution.^2^ Furthermore, in its current state, QCEIMS only allows for singly ionized species.

### 2.2 GFN-xTB and IPEA-xTB

The GFN-xTB method was developed and published very recently in our laboratories, and to familiarize the reader with the method, we give a brief but essential introduction to the features and ideas of GFN-xTB below, along with a short description of IPEA-xTB which is founded on the GFN-xTB method. For a more in-depth discussion the reader is referred to ref. 43. The GFN-xTB is a special-purpose semi-empirical approach analogous to the well-established DFTB3 method.^50^ The GFN-xTB is motivated from the success of its predecessor sTDA-xTB,^51–53^ where an extended TB scheme is used to calculate, with good accuracy, electronic excitation spectra of large molecules. The new modified extended TB variant GFN-xTB, targets geometries, frequencies and non-covalent interactions (hence the namesake, “GFN”). It has been shown to yield more accurate results for the given target properties than other general semi-empirical methods, which usually attempt to capture both structural and energetic features (*e.g.*, thermochemistry) simultaneously.^43^ The method is described as extended (denoted by “x” in xTB) because it employs partially polarized minimal basis sets, *i.e.*, with an additional s-function on H and d-functions for third row and higher elements. The use of an extended basis set largely alleviates problems in describing systems with polar bonding *e.g.*, hydrogen and hypervalent bonding situations for heavier elements. Furthermore, the GFN-xTB is found to be computationally faster than other comparable methods mainly due to quick and robust convergence of the electronic iterations.^43^ Therefore, large-scale quantum chemical treatments of complex molecular systems can be performed routinely. The number of empirical method parameters is minimized and restricted to global and element-specific values, making it more transferable and easy to parametrize. There are only 19 global parameters and approximately 10 element specific parameters included in the GFN-xTB method. The parameters have been fitted to hybrid DFT data, where the target quantities are equilibrium and slightly distorted structures, harmonic vibrational frequencies, CM5 atomic charges^54^ and non-covalent interactions energies and structures. Currently, parameters exist for elements up to *Z* = 86, making the method applicable to a large range of chemical systems. The aforementioned properties: fast computations, robustness and wide applicability of GFN-xTB along with precise analytical nuclear gradients make the approach ideal to use in conjunction with QCEIMS. In the xTB approach, the total energy is expressed as a sum of four terms; the electronic energy (*E*_el_), the repulsion energy (*E*_rep_), the well-known D3(BJ)^55–57^ dispersion energy (*E*_disp_), and a classical correction for halogen-bonding interactions (*E*_XB_). The electronic energy is computed by a SCC treatment, analogous to that of DFTB3. For a derivation and details of the GFN-xTB method, see ref. 43. As discussed in the original publication, the use of a finite electronic temperature treatment^58–60^ (see below) allows proper dissociation of covalent one- and two-electron chemical bonds which is of vital importance for QCEIMS.

The second TB variant, IPEA, is also of special-purpose and succeeds from the GFN-xTB. It is a straightforward re-parametrization to calculate reasonably accurate IPs and EAs up to a constant empirical shift. Moreover, the IPEA variant uses additional (*n* + 1)s basis functions. It has been re-fitted to reference IP/EA values for parts of the original GFN-xTB training data set. The reference IP/EA values are computed by PW6B95/def2-TZVPD^61^ with TURBOMOLE 7.1.^34,62^ Typical errors for computed vertical IP/EA values by IPEA-xTB compared to DFT are 0.2–0.4 eV. Both the GFN and IPEA parametrizations used by QCEIMS are available from the authors^46^ and a more detailed discussion of the accuracy of IPEA-xTB for IP/EA will be given elsewhere in the context of electrochemistry applications. At this point, the special IPEA-xTB parameters are only available for parts of the periodic table, excluding the transition metals. For such species the standard GFN-xTB element parameters are used for the IP calculation step and we present MS(GFN-xTB) acquired spectra for the organometallic complexes in the ESI.† As discussed above, in such cases MS(GFN-xTB/DFT) should currently be used and ongoing work in our group is devoted to cover all elements by IPEA-xTB.

The single point calculations involved in the first two steps of the QCEIMS protocol (equilibration, sampling and the MO spectrum calculation) and also the IP evaluations employ Fermi-smearing^58^ at a default electronic temperature of 300 K. In the regular classical propagation of the nuclei, during the third step of QCEIMS (*i.e.*, production runs), the electronic temperature is chosen to be 5000 K (*cf.* the ESI of ref. 2). Fermi smearing is found to facilitate SCC convergence and partially remedy electronically complicated situations which arise during the MD trajectories. It is essential to qualitatively describe the dissociation of the parent ion and fragments to (radical) neutral and charged moieties, without resorting to impracticable multi-reference theory.

### 2.3 Performance

The production runs are executed in parallel on Intel(R) Xeon(R) E5-2660 2.00 GHz cores, where each production run occupies only a single core. The total number of MS(GFN-xTB) single point energy/gradient calculations performed in the production runs of all included test set molecules surmounts to roughly 270 million. This sheer number emphasizes the need to use incredibly efficient electronic structure methods in conjunction with QCEIMS.

**Fig. 3:**
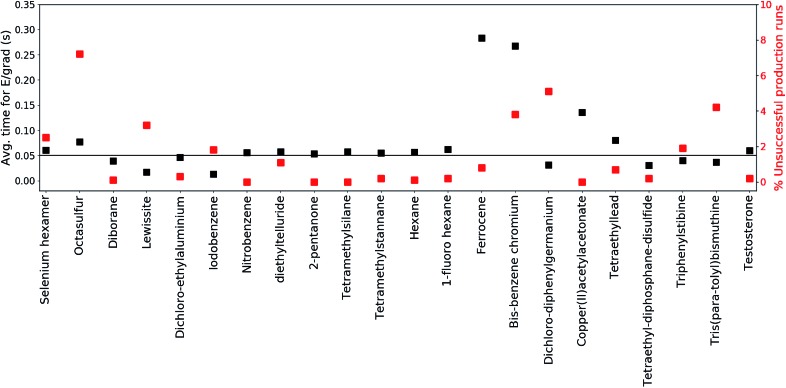
The average time per single energy/gradient computation and robustness is depicted for all of the test-set molecules. The molecules are listed on the vertical axis, by ordering of increased molecular size (left to right). The average computational time is depicted on the left vertical axis and denoted by black squares. The robustness (percentage of unsuccessful production runs out of 1000) is depicted on the right vertical axis and denoted by red squares. **10** is an outlier and is omitted for clarity.

To further inspect the computational speed and robustness of MS(GFN-xTB) (and MS(GFN-xTB/DFT)), the average time per energy/gradient computation and the percentage of unsuccessful production runs is reported for each molecule in Fig. 3, with the exception of **10** (which is specifically addressed below). The average time per computation is found to be roughly 0.05 seconds, *i.e.*, 20 force evaluations per second, for both the main group inorganic and organic group molecules. The average time per energy/gradient computation for the organometallic molecules, is found to range from 0.15 to 0.30 seconds. The one order of magnitude increase in the computational time, from organic to organometallic molecules, is ascribed mainly to the overhead by the hybrid DFT IP evaluations. Moreover, multiplicity checks are employed for all fragments that include 3d transition metals, increasing the number of energy evaluations per IP. In the special case of **10**, we find the average time to be approximately 1.4 seconds per calculation resulting from the increased DFT overhead of the relatively large molecule.

It is hard to generalize about the wall-time required to simulate a EI-MS beforehand, since the computational time is heavily influenced by the input molecule itself, the number of production runs, cascades and fragments, and available computational resources. For this purpose we have listed the average and maximum number of energy/gradient computations required by the production runs, as well as, the computational times in the ESI.† We find that the average number of energy/gradient computations per production run ranges from roughly 4000 (**12**) to 15 000 (**19**) calculations. For the organic and main group inorganic molecules, the average wall-time per production run, can range from roughly 86 (**18**) to 857 (**6**) seconds. For the organometallic molecules the average wall-time per production run ranges from 1630 (**9**) to 18 700 (**10**) seconds.

The robustness of MS(GFN-xTB) is found to be remarkably good. The majority of the molecules exhibit less than 2% unsuccessful production runs. The number of unsuccessful runs for **10** is found to be exactly 4% and only three molecules (**14**, **20** and **21**) have >4% failure rate. For these three molecules, the number of unsuccessful runs is between 5 and 7% which we consider as borderline for an unbiased sampling. In case of higher failure rates one would increasingly sample merely the electronically ‘simple’ part of the reaction space leading to biased results.

In order to predict relatively accurate theoretical EI-MS for an almost arbitrary molecule, the PES of the GFN-xTB has to parallel the ‘true’ PES for a wide range of nuclear arrangements. Therefore, we inspect a few simple exemplary reaction coordinates for decomposition pathways occurring in our simulations, using hybrid FT-DFT as reference, which are given in the ESI.† Exemplary dissociation curves are additionally discussed in the original GFN-xTB publication.^43^ Analysis of the data shows that potential energy curves for simple dissociation (using GFN-xTB) are relatively accurate despite the fact that the method was not primarily parametrized for energetic properties. We attribute this success (and that of the entire MS(GFN-xTB) scheme) to the inherent ability of TB methods to properly dissociate bonds in tandem with our specific fit to vibrational frequencies (yielding accurate force constants) and also to Fermi smearing.

## 3 Results and discussion

**Fig. 4:**
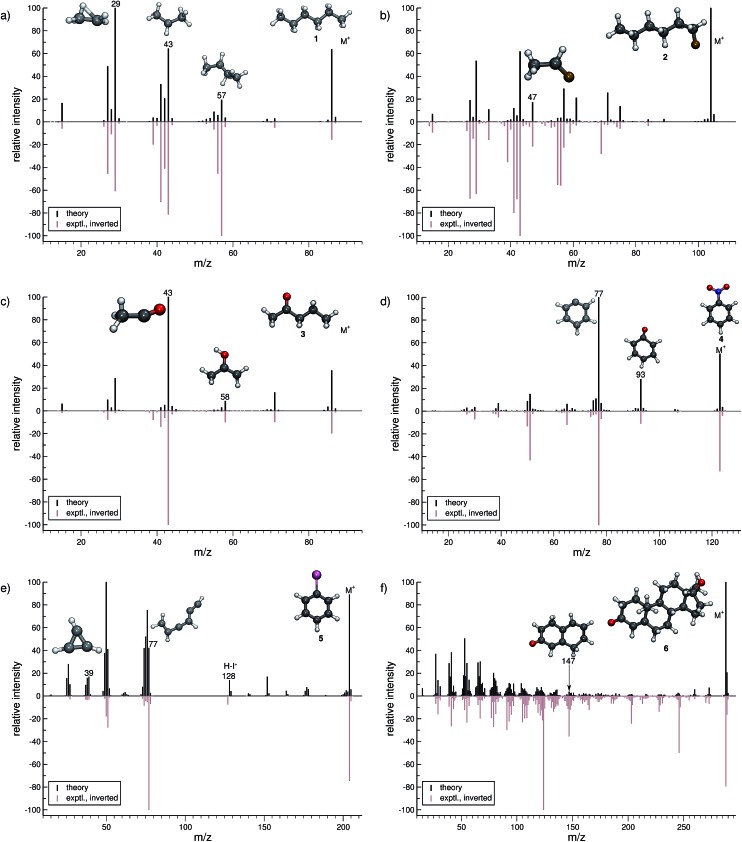
Comparison of computed and experimental EI-MS for the organic set, including molecules **1–6** in (a–f), respectively. The structures of the parent ion (denoted by M^+^) and selected ionic fragments have been superimposed on each computed spectrum. Moreover, the selected ions are marked by the respective *m*/*z* values and discussed in the text.

**Fig. 5:**
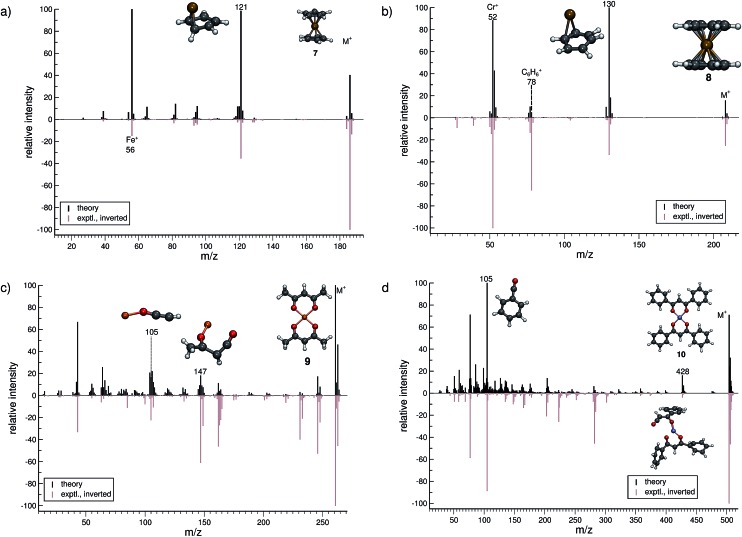
Comparison of computed and experimental EI-MS for the organometallic group, including molecules **7–10** in (a–d), respectively. The structures of the parent ion (denoted by M^+^) and selected ionic fragments have been superimposed on each computed spectrum. Moreover, the selected ions are marked by the respective *m*/*z* values and discussed in the text.

**Fig. 6:**
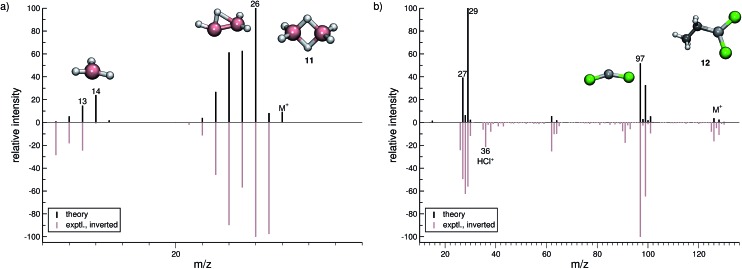
Comparison of computed and experimental EI-MS for group 13 inorganic molecules, **11** and **12** in (a and b), respectively. The structures of the parent ion (denoted by M^+^) and selected ionic fragments have been superimposed on each computed spectrum. Moreover, the selected ions are marked by the respective *m*/*z* values and discussed in the text.

**Fig. 7:**
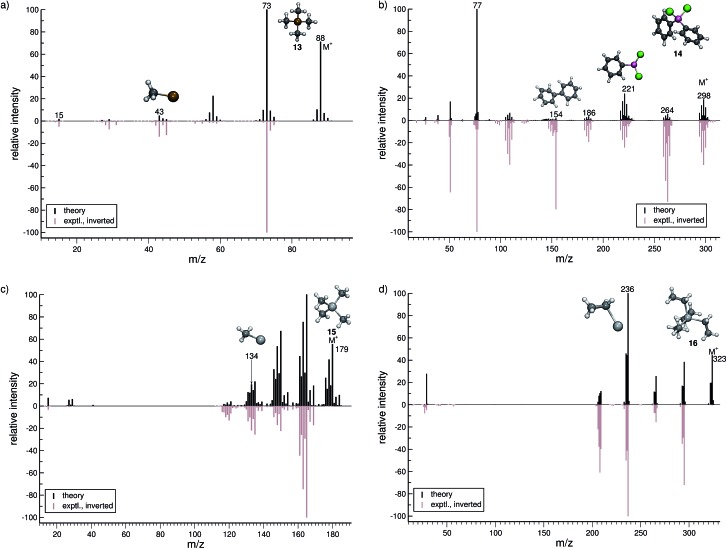
Comparison of computed and experimental EI-MS for group 14 inorganic molecules **13–16** in (a–d), respectively. The structures of the parent ion (denoted by M^+^) and selected ionic fragments have been superimposed on each computed spectrum. Moreover, the selected ions are marked by the respective *m*/*z* values and discussed in the text.

**Fig. 8:**
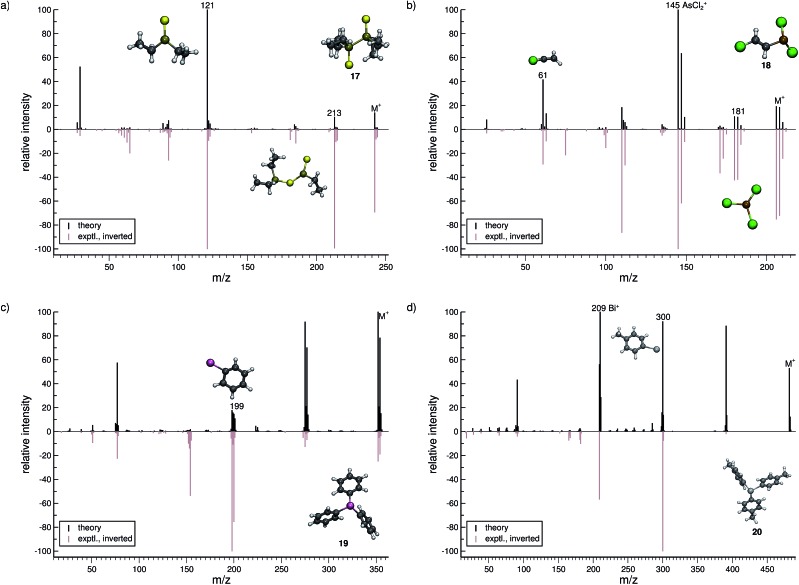
Comparison of computed and experimental EI-MS for group 15 inorganic molecules, **17–20** in (a–d), respectively. The structures of the parent ion (denoted by M^+^) and selected ionic fragments have been superimposed on each computed spectrum. Moreover, the selected ions are marked by the respective *m*/*z* values and discussed in the text.

**Fig. 9:**
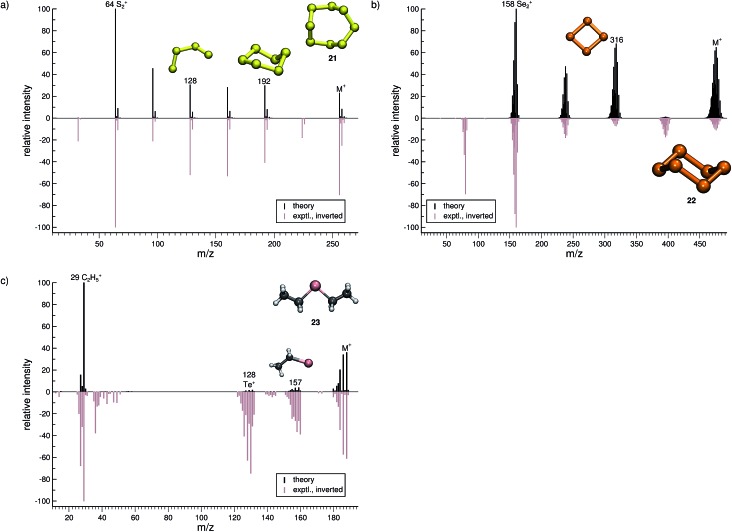
Comparison of computed and experimental EI-MS for group 16 inorganic molecules, or molecules **21–23** in (a–c), respectively. The structures of the parent ion (denoted by M^+^) and selected ionic fragments have been superimposed on each computed spectrum. Moreover, the selected ions are marked by the respective *m*/*z* values and discussed in the text.

In this section, we present simulated EI-MS for all molecules of the test set (**1–23**) and compare the results directly to the respective experimental spectra. We address some fragment structures, investigate reaction pathways with an emphasis on molecular rearrangements and perform fragment-to-peak assignments for chosen signals, *e.g.*, determinative peak-series. In the analysis of the production run trajectories, we use the same script as previously reported^12^ to identify fractional yields and distinguish between structural isomers that contribute to the same peak, or mass-to-charge ratio *m*/*z*. The comparison of the experimental and computed MS, for all molecules, is visualized in Fig. 4–9. The molecular ion and ionic fragments, which are discussed in the text, have been superimposed on the computed MS depicted in the figures. The visualized structures are taken as the average fragment structures over the last 50 MD steps in the production run trajectories and are also labeled by their *m*/*z* values.

### 3.1 Organic molecules (**1–6**)

For hexane (**1**, Fig. 4a), the simulated MS is found to be in very good agreement to the experimental spectrum, where the observed peak series *m*/*z* 57 (C_4_H_9_^+^), *m*/*z* 43 (C_3_H_7_^+^), and *m*/*z* 29 (C_2_H_5_^+^) is reproduced by the simulations. Moreover, we find the parent ion to be slightly too stable, in the simulations. An inspection of the trajectories reveals that fragments *m*/*z* 58 and *m*/*z* 43 result from the formation of a tertiary cation, where a H atom migrates to a terminal position. Moreover, the simulations successfully predict the fragment *m*/*z* 29 to have the confirmed ‘non-classical’ ethyl cation structure.^63,64^

For 1-fluorohexane (**2**, Fig. 4b), the experimental and computed MS are in good agreement. However, the parent ion does not decompose in a large number of production runs, meaning the survival rate of the **2** cation is too high under the given simulation conditions. This problem can be partially alleviated by applying a higher IEE and/or longer simulation times. We would like to stress that the IEE distribution is not obtained specifically by *ab initio* QC calculations, but rather assumed to be a Poisson type variant, for all molecules. Therefore, such effects are to be expected for certain molecules with unusual (1e–2e)–EI cross sections. The same determinative peak series, as observed in the MS of **1**, is observed for **2** and is again reproduced. Moreover, an additional signature peak is observed for **2**. It corresponds to the fragment *m*/*z* 47 (C_2_H_4_F^+^) and involves a H atom migration, to form the more stable cation.

The next molecule is 2-pentanone (**3**, Fig. 4c). This molecule is of a particular difficulty,^2^ since the parent ion can undergo the well-known McLafferty rearrangement.^65,66^ This reaction is characterized by an H atom transfer to the carbonyl oxygen and a subsequent loss of a neutral olefin molecule, or propylene in our case. Indeed, the correct ion, *m*/*z* 58 (C_3_H_6_O˙^+^), is reproduced in the simulations. Moreover, the base peak is correctly computed to be *m*/*z* 43 (C_2_H_3_O^+^) and is found to result from α-cleavage. Overall, the experimental and computed MS of **3** are in excellent agreement.

The simulated MS of nitrobenzene (**4**, Fig. 4d) contains all of the statistically significant peaks, found in the experimental spectrum. The relative intensity of the parent ion signal is reproduced quite well. The fragment *m*/*z* 93 (C_6_H_5_O^+^) is an example of how complex molecular rearrangements are captured by the simulations. Here, the fragment can only form after loss of an NO molecule, subsequent to an oxygen atom migration. Moreover, the fragment *m*/*z* 77 (C_6_H_5_^+^) is the cyclic phenyl cation and it forms the base peak in both the computed and experimental MS. We find the fractional yield of this phenyl cation to be 33%. The agreement between the simulated and experimental spectra of **4** is good.

The phenyl cation is also one of the main products in the fragmentation of iodobenzene (**5**, Fig. 4e). Interestingly, an analysis of the fragment *m*/*z* 77 reveals that the cyclic phenyl cation has a fractional yield of only 3.7%, whereas various acyclic isomers of C_6_H_5_^+^ are formed as well. This is in line with experimental studies that find a certain fraction of acyclic C_6_H_5_^+^ in IR measurements, following the dissociation of halobenzenes.^67^ Another peak that occurs in the MS of both **4** and **5** is *m*/*z* 39 (C_3_H_3_^+^) which has the structure of the cyclopropenyl cation.^68^ The comparison between the computed and experimental MS of **5** is reasonable, as there are a few artifacts observed in the computed MS. Additionally, certain peaks are found to be over pronounced *e.g.*, *m*/*z* 128 (H–I˙^+^), in the computed spectrum.

The MS of testosterone (**6**, Fig. 4f) contains a large number of peaks, and many, but not all of them are found in the computed MS. Most fragmentation pathways of **6** are underestimated by the simulations, even though the peak series in the lower-mass end of the spectrum is reproduced quite well. The stability of the parent ion appears to be estimated accurately. We have picked only one isomer contributing to the peak *m*/*z* 147 (C_10_H_11_O^+^) to be displayed in Fig. 4f. As there are only a few production runs that yield this particular ion, it is not clear whether the displayed structure is the most abundant isomer. However, this structural isomer results from the cleavage of two rings of the steroid scaffold, which appears to be a reasonable pathway.

In summary, the computed EI-MS for the organic group, **1–6**, are in general found to compare very well with the experimental spectra, using the new MS(GFN-xTB) approach. The quality of the spectra is comparable or slightly better than previous results acquired using other similar semi-empirical methods.^40^ Furthermore, we show that the simulations are able to shed light on complex dissociation dynamics, where molecular rearrangements occur naturally in the simulation trajectories, *e.g.*, the McLafferty rearrangement for **3** and an oxygen atom transfer for **4**. Additional computed EI-MS of organic molecules have been included in the ESI.†

### 3.2 Organometallic molecules (**7–10**)

The treatment of organometallic molecules is challenging because of their more complicated electronic structures already in the neutral ground state.

The computed MS of ferrocene (**7**, Fig. 5a) compares well to the experimental spectrum, which is a big success of the new approach. There are a number of peaks that are clearly over-pronounced in the computed spectrum, which can partially be attributed to the too low stability of the parent ion. The parent ion is not found to be the base peak in the computed spectrum. Instead, the base peak, *m*/*z* 121, is the C_5_H_5_Fe^+^ fragment, which is formed by the loss of one cylopentadienyl ligand from the parent ion. Fe^+^ (*m*/*z* 56) is observed, both experimentally and in the computed spectrum. The percentage of failed runs is only about 1%, which is remarkable considering the electronic complexity of the ferrocene radical cation in particular.^69^

There are no major artifacts found in the computed MS of bis-benzene chromium (**8**, Fig. 5b). We consider the comparison between the experimental and computed spectrum to be good. We find the ion *m*/*z* 130 (formed by benzene loss from the parent ion), the benzene cation *m*/*z* 78, the chromium ion *m*/*z* 52 and a few less intense peaks. However, the latter cannot be considered representative, since the number of production runs corresponding to these peaks is smaller than the number of failed production runs, which is 3.8%.

The computed spectrum of copper(ii)acetylacetonate (**9**, Fig. 5c) compares adequately to the experiment. Most of the peaks are reproduced in the simulations. However, there are a few artifacts in the simulated spectrum, but the extent of these is small. The fragment *m*/*z* 147 (C_4_H_4_O_2_Cu^+^) forms by the loss of an acetylacetonate ligand and a methyl radical. Moreover, the fragment *m*/*z* 105 (C_2_HOCu˙^+^) requires even more bond ruptures and results from the *m*/*z* 147 fragment *via* carbon monoxide and methyl radical loss. Comparable to **7**, the number of unsuccessful production runs is below 1%.

The nickel(ii)bis(diphenyl-acetylacetonate) (**10**, Fig. 5d) molecule is the largest and in many ways the most challenging system, investigated in this study. This is reflected by the higher percentage failure rate of 4.0%. Nevertheless, the computed MS compares moderately well to the experimental spectrum, where a number of peaks are successfully reproduced, *e.g.*, the fragment *m*/*z* 428 (C_24_H_17_O_4_Ni^+^), which forms after a phenyl radical loss and also the benzoyl cation at *m*/*z* 105 (C_7_H_5_O^+^). We stress that quantum chemical calculations on nickel complexes have remained a major challenge for DFT. Therefore, the computation of the EI-MS of **10** stands out among the results, even if the agreement between theory and experiment is not quantitative.

In summary, the comparison of experimental and computed MS for the organometallic molecules, **7–10**, provides us with the confidence that unimolecular decomposition pathways of cationic transition metal complexes can indeed be studied, in detail, with the novel MS(GFN-xTB/DFT) combination. This can also be seen from further computed MS shown in the ESI.† The unprecedented success indicates the quality and robustness of the underlying GFN-xTB method, a really intriguing finding that could not be fully anticipated from its construction principle.^43^ For organometallic complexes, we advocate at this point the use of hybrid DFT for the calculation of IPs, where reasonably accurate IP calculations play the central role in determining the computed peak intensities. As discussed in Section 2, even the relatively few DFT calculations will become the computational bottleneck of the whole procedure. Improving the preliminary parametrization of IPEA-xTB for organometallic compounds (which can be considered as a worst case scenario for the entire QCEIMS) might resolve this issue. It is in any case very encouraging to see the possibility of realistic theoretical EI-MS for organometallic compounds without any significant modifications or empirical adjustments of the procedure.

### 3.3 Inorganic molecules (**11–23**)

#### 3.3.1 Group 13 (**11–12**)

For diborane (**11**, Fig. 6a), the comparison of the simulated and experimental MS is good. The fragmentation cascades consist of multiple hydrogen losses, both in the form of single H atoms and H_2_ molecules. The base peak is correctly computed to be the B_2_H_4_˙^+^ (*m*/*z* 26) fragment. We observe an interesting structure for this fragment, ascribed to the H atom mobility in the diborane cation where the H atoms can move freely between the boron centers. The fragment BH_3_^+^ (*m*/*z* 14) forms by a rupture of the boron–boron bond. However, the BH_3_^+^ fragment is found to be less abundant than fragments of *m*/*z* 13 and *m*/*z* 12 in the experimental MS, showing that H atom loss continues even after the B–B bond rupture. This is also reflected in the simulated spectrum.

In the case of dichloro-ethylaluminium (**12**, Fig. 6b), the experimental and computed spectra are in a somewhat poorer agreement, where several peaks observed experimentally are missing in the computed spectra. The dominant reaction pathway is the dissociation of the ethyl moiety from the parent ion, resulting in the formation of AlCl_2_^+^ (*m*/*z* 97) and an ethyl cation (*m*/*z* 29). The IPs of these ions are comparable, which is why both ions are observed in the simulated spectrum. We note that HCl˙^+^ is observed in the experimental MS of **12**. This fragment will be assigned a negligible statistical charge in the QCEIMS procedure because of the large IP of HCl. In the experiment the HCl˙^+^ fragment may form from a reaction involving **12** and H_2_O, prior to the ionization of **12**. For volatile compounds, such peaks can be rationalized too by our procedure.

#### 3.3.2 Group 14 (**13–16**)

The computed MS of tetramethylsilane (**13**, Fig. 7a) compares well to the experimental MS, although the computed survival rate of the parent ion is much too high. The base peak, assigned to the fragment *m*/*z* 73 (C_3_H_9_Si^+^) is reproduced in the computed MS. This fragment is formed by the loss of a methyl group, from the parent ion. Interestingly, there is a very weak signal for the double methyl loss in the experiment, while the same signal is predicted to be strong in the computed spectrum. The fragment CH_3_Si^+^ (*m*/*z* 43) is correctly computed to have a relatively low abundance. In addition, in the MS of **13**, the importance of the statistical charge model becomes even more evident for the observed methyl ion (*m*/*z* 15) signal. The methyl cation acquires a non-negligible statistical charge because of the higher but still relatively similar IP of the methyl radical compared to the other reaction species.

For dichloro-diphenylgermanium (**14**, Fig. 7b), the computed and experimental MS are in relatively good agreement. The peak series (*m*/*z* 264, 221, 186) reflects the loss of one chlorine atom, one phenyl group and one phenyl group as well as two chlorine atoms, respectively. The IP of the phenyl fragment (*m*/*z* 77) is relatively low, and consequently, it is computed to be the base peak. Here, the formation of the biphenyl cation, C_12_H_10_˙^+^ (*m*/*z* 154) is observed. This interesting reaction pathway is underrepresented in the production runs with a fractional yield of only 0.7%. It proceeds by a molecular rearrangement, followed by a C–C bond formation.

The computed MS of tetramethylstannane (**15**, Fig. 7c) compares very well to the experimental MS. The decomposition of the parent ion is governed by a series of methyl losses, resulting in the formation of *m*/*z* 164 (C_3_H_9_Sn^+^), 149 (C_2_H_6_Sn^+^), 134 (CH_3_Sn^+^) and the naked Sn^+^ (*m*/*z* 119) cation. This peak series is accurately captured by the simulations. The parent ion is observed to be statistically insignificant in the experiment, whereas we find it to be a relatively intense signal theoretically. This discrepancy is attributed to the high stability of the parent ion in the simulations which can be improved by adjusting the IEE simulation parameters as noted above.

For tetraethyllead (**16**, Fig. 7d), the agreement between experiment and simulation is good. The MS reflects a series of ethyl losses, and the base peak is accurately predicted to be the C_2_H_5_Pb^+^ (*m*/*z* 236) fragment. As for **15**, the peak corresponding to the parent ion is negligible in the experimental MS, while being a relatively intense signal computationally.

#### 3.3.3 Group 15 (**17–20**)

Tetraethyl-diphosphane-disulfide (**17**, Fig. 8a) exhibits an interesting isomerization reaction subsequent to an ethyl radical loss from the parent ion, yielding the C_6_H_15_P_2_S_2_^+^ (*m*/*z* 213) fragment. As visualized in Fig. 8a, this fragment no longer has a P–P bond, instead the sulfur atom rearranges to form a bridging P–S–P bond. The base peak is correctly found to be the fragment C_2_H_10_PS^+^ (*m*/*z* 121) and results from a rupture of the P–P bond, in which the phosphorous adopts a trigonal coordination. The overall agreement between the spectra is decent, where the molecular ion is predicted to be slightly too unstable compared to the experiment.

Lewisite (**18**, Fig. 8b) is used as a chemical weapon and hence it is important to understand its MS and that of its derivatives, for analytical purposes.^70^ We find the overall comparison between the experimental and simulated spectra to be good. One interesting reaction is the formation of the AsCl_3_˙^+^ (*m*/*z* 181) fragment. The pathway is observed in a number of trajectories, where it proceeds by a 1,3-chlorine atom shift in the parent ion. The most abundant fragment (base peak) is found to be AsCl_2_^+^ (*m*/*z* 145), in both the computed and experimental spectrum. The structure of the C_2_H_2_Cl^+^ (*m*/*z* 61) fragment results from yet another case of H atom migration, where an H atom is transferred to the terminal carbon atom of the fragment.

For triphenylstibine (**19**, Fig. 8c), we observe several artifacts in the computed MS, primarily of low abundance. The dominant peak series in the MS corresponds to the parent ion and subsequent dissociation of phenyl groups. The base peak of the computed spectrum is found to be the parent ion, while the experimental base peak corresponds to the fragment C_6_H_5_Sb˙^+^ (*m*/*z* 199), *i.e.*, the survival rate of the parent ion is too high in the simulations. However, we find the *m*/*z* 199 peak to be statistically significant in the computed spectrum, even if it is not the main peak. The quality of the calculation in this case, may be considered as mediocre. However, it is still useful for molecular identification because the computed spectra accurately captures the characteristic peak series, of subsequent phenyl group losses.

For tris(*para*-tolyl)bismuthine (**20**, Fig. 8d), the two spectra do not compare so well, with numerous low-intensity artifacts found in the computed spectrum. The parent ion is absent in the experimental spectrum, while being a relatively large signal theoretically. The characteristic peak series is represented by subsequent tolyl losses from the parent ion, and this peak series is reproduced in the computed spectrum. However, the first peak in the series has a much larger intensity in the computed than in the experimental spectrum. The second peak (*m*/*z* 300) forms by the loss of two *para*-tolyl groups, yielding the C_6_H_5_Bi˙^+^ fragment. It is observed as the base peak in the experimental spectrum, while the last peak of the series is predicted to be the base peak in the computed spectrum. This peak corresponds to the naked Bi^+^ cation, where the parent ion has lost all of the *para*-tolyl substituents. We honestly include (**20**) as an example of cases with relatively bad correspondence between theory and experiment. If this is rooted in an inaccurate parametrization of bismuth (in GFN-xTB) or related to some other problem, specific to very heavy elements, it will have to await further investigation.

#### 3.3.4 Group 16 (**21–23**)

For the most prevalent allotrope of sulfur, cyclic S_8_ (**21**, Fig. 9a), the agreement between the experimental and computed spectra is found to be good. However, there are two peak-signals missing in the computed spectrum, the ion formed after a loss of a single S atom (*m*/*z* 226) and the S^+^ ion (*m*/*z* 32), which most likely are complementary to one another. The remaining signals are captured by the simulations, where the fragment S_6_˙^+^ (*m*/*z* 192) adopts a cyclic structure and S_4_˙^+^ (*m*/*z* 128) is an open-chained structure. The base peak in the MS of (**22**) is correctly found to be *m*/*z* 64, corresponding to the S_2_˙^+^ fragment ion.

The quality of the simulation for Se_6_ (**22**, Fig. 9b) is comparable to that of **21**. The ion formed after a single Se atom loss (*m*/*z* 395) and the Se^+^ ion (*m*/*z* 79) are underrepresented and missing in the simulations, respectively. The fragment Se_2_˙^+^ (*m*/*z* 158) is correctly found to be the base peak in the computed spectrum. Interestingly, the fragment *m*/*z* 316, Se_4_˙^+^, may assume a cyclic structure based on the visualization of the trajectories. This reaction could be investigated at a higher level of theory. Overall, the computed and experimental MS are in reasonable agreement.

The final molecule of this study is diethyltelluride (**23**, Fig. 9c). We observe a moderate agreement between the computed and experimental MS. Numerous peaks are missing in the computed MS *e.g.*, the fragments observed with *m*/*z* around 40 and 142. What is more, the intensities of few signals are drastically underrepresented. However, the intensity of the parent ion is correctly computed. Moreover, the simulations are able to reproduce the base peak *m*/*z* 29, which corresponds to the fragment C_2_H_5_^+^, as well as the naked Te^+^ (*m*/*z* 128).

For the main group inorganic molecules, **11–23**, the computed MS generally compare relatively well to the experimental spectra. The main peak series is usually fully reproduced by the simulations, even though the intensities can be somewhat inaccurate. We consider the applicability of the MS(GFN-xTB) combination to be evident from the computed EI-MS and think that the method can be convincingly applied to a large variety of molecular systems, comprising main group elements. Of course, the quality and furthermore the faults of the computed EI-MS will differ from one molecule to another, *e.g.*, the parent ion is predicted to be too stable for molecules **13**, **15**, **16**, **19** and **20**, several artifacts are found in the MS of **19** and **20**. The computed MS of **12**, **21**, **22**, **23** have a few missing peak-signals. It is important to note that all of the EI-MS are simulated using fixed conditions and nothing has been ‘cherry-picked’. In general the quality of the spectra can be slightly improved by varying the simulation conditions for each case (mainly average IEE and simulation time).

## 4 Conclusions

We have implemented the recently developed, special-purpose, GFN-xTB and IPEA-xTB semi-empirical methods in QCEIMS, making QCEIMS fully operational without inclusion of any third-party software. It is now applicable to molecules composed of elements with atomic numbers up to *Z* = 86. The methods are devised to accurately compute *e.g.*, atomic forces and IPs, respectively, in a computationally efficient manner. The main method GFN-xTB which provides the PES for all occurring reactions was not modified for the present purpose. Because of their robustness and computational efficiency, GFN-xTB and IPEA-xTB are ideal to use in conjunction with QCEIMS. To evaluate the performance and transferability of MS(GFN-xTB), we have simulated EI-MS for 23 chemically diverse molecules. The molecules are divided into three groups comprising of organic (**1–6**), organometallic (**7–10**) and main group inorganic molecules (**11–23**). Such extensive quantum chemistry calculations of EI-MS for molecules across the periodic table is unprecedented.

There were roughly 270 million single point energy and gradient calculations conducted for this study, using the GFN-xTB method (the number of IP evaluations is included in the count, but is negligible). We find GFN-xTB to be remarkably robust with typically less than 2% unsuccessful production runs. Furthermore, because of its good convergence properties, GFN-xTB is extremely fast, where MS(GFN-xTB) performs, on average, around 20 energy/force evaluations per (real-time) second, irrespective of the given molecular size (up to 49 atoms as in **6**) and composition. It is evident that MS(GFN-xTB) is both robust and computationally efficient, enabling exhaustive simulations of EI-MS for the first time.

For the organic molecules, the MS(GFN-xTB) computed MS compare generally well to the respective experimental spectra. The GFN-xTB computed spectra are of comparable quality to those published previously for similar compounds, using related semi-empirical methods in tandem to QCEIMS.^40^ The visualization of the simulation trajectories reveals a number of interesting reaction pathways. As an example, for **1** and **2**, H atom migration to a terminal carbon atom is observed. For **3** the simulations are able to reproduce the McLafferty rearrangement and for **4**, an oxygen atom is transferred to a carbon atom, prior to NO loss. Interestingly, the peak-signal *m*/*z* 77 is observed in the MS of both **4** and **5**. For **4** this peak is solely ascribed to the cyclic phenyl cation (*m*/*z* 77). However, for **5** the peak results from various acyclic isomers of C_6_H_5_^+^ and the cyclic phenyl cation. We conclude that one can convincingly simulate electron ionized fragmentation pathways of organic radical cations using MS(GFN-xTB).

The novel prediction of EI-MS of the organometallic molecules is achieved at the typical speed of semi-empirical QC calculations, for the first time. The quality of the computed MS of **8** is striking considering the complexity of the problem. The quality of the spectra of **7**, **9** and **10** is not as good, but can be considered satisfactory. For **7** the parent ion is found to be too unstable in the simulations, resulting in over pronounced fragment peak intensities. Furthermore, the spectra for the latter three molecules exhibit a few artifacts, or false-positive peak-signals. Nevertheless, the accuracy which MS(GFN-xTB) attains in the prediction of EI-MS for organometallic molecules is hard to achieve, even by simulations conducted using standard DFT methods. We hold that the quality of the MS is sufficient to enable investigations into the various fragmentation pathways of organometallic cations. Adjustments of the electronic parameters in the GFN-xTB Hamiltonian in particular for the transition metal complexes could further improve the quality of the theory. We stress that the fragment IPs play a pivotal role in the QCEIMS procedure, where they are used to determine the statistical charges and hence the peak intensities. Therefore, in the case of electronically complicated transition metal complexes, we advocate the use of hybrid DFT for the computations of IPs. On the downside this can drastically increase the overall simulation time, depending on the molecule under study.

The computed EI-MS of the inorganic main group molecules (**11–23**), further attests to the transferability and accuracy of the MS(GFN-xTB) approach across the periodic table. Generally, the computed and experimental spectra compare relatively well. Therefore, the procedure allows for an unprecedented and unbiased insight into the fragmentation pathways of inorganic main group molecules. As an example, the simulations are able to capture many interesting reaction pathways *e.g.*, the formation of the biphenyl cation from **14**, the rearrangement and formation of a P–S–P bond subsequent to an ethyl radical loss from **17** and 1,3-chlorine atom shift of the cation of **18** required to form the AsCl_3_˙^+^ fragment. The worst agreement between the computed and experimental spectra is observed for **12** and **23**. Also, for **23** some of the peak intensities are severely underestimated. We find that for alkylated and arylated compounds (**13**, **15**, **16**, **19**, **20**) the parent ion appears to be artificially too stable in the simulations, ascribed to an interplay of the simulation time and IEE.

Indeed, the typical errors in a computed MS are missing peaks, inaccurate intensities, artifacts and too high stability of the molecular parent ion. In most cases the MS can be improved by varying the simulation conditions, until an optimum spectrum is produced. More importantly, the implementation of GFN-xTB and IPEA-xTB (in QCEIMS) allows for further improvements to an individual MS, where the methods can be easily be re-parametrized to high-level reference data, for the system of interest. This direction and its impact on the quality of spectra will be explored in forthcoming work.

It has to be kept in mind that GFN-xTB and IPEA-xTB are semi-empirical methods and thus retain the fundamental deficiencies introduced by, *e.g.*, the parametrization, integral approximations and small basis sets. Therefore, it is to be expected that the MS(GFN-xTB) approach fails for some systems. Examples where the theoretical MS are of unacceptable quality, are given in the ESI.† We also compare MS(GFN-xTB) to the semiempirical DFTB3-D3 and PM6-D2H^21,71^ PES in three illustrative cases in the ESI,† where GFN-xTB has given clearly superior results. We submit that the apparent accuracy of GFN-xTB for the majority of cases stems from error cancellation of systematically too deep potential wells leading to high barriers and the inherent TB self-interaction error which works in the opposite direction. Preliminary results comparing the GFN-xTB to high-level *ab initio* PES have been obtained and will be presented in a forthcoming report.

Nevertheless, the surprisingly high quality of the simulated EI-MS enables a fast overview of the unimolecular fragmentation space for a wide variety molecules. On these terms, one important aspect of MS(GFN-xTB) is the screening of possible reaction pathways, which are then later refined at a higher level of theory, thereby, avoiding prior knowledge (or assumption) of reaction channels. This may lead to the discovery of new reaction types and the elucidation of reaction mechanisms, especially concerning gas phase ion chemistry of transition metal complexes, for which DFT calculations are in high demand.^35–39^

The expansion of QCEIMS to simulations of electrospray ionization/collision induced dissociation (ESI/CID) mass spectrometry, techniques, where the initial conditions for an MD based theoretical QC treatment are more well-defined than for EI-MS, is underway in our laboratory.
