# Supplementary material for: Quantum chemical calculation of electron ionization mass spectra for general organic and inorganic molecules
Source: Chem Sci. 2017 May 5;8(7):4879–95. doi: 10.1039/c7sc00601b (PMC5603848; doi:10.1039/c7sc00601b)
Supplement: Supplementary file 1 [file SC-008-C7SC00601B-s001.pdf]

Electronic Supplementary Material (ESI) for Chemical Science.
This journal is © The Royal Society of Chemistry 2017

Quantum Chemical Calculation of Electron Impact Mass Spectra for General Organic and Inorganic Molecules

–

Electronic Supplementary Information

Vilhjálmur Ásgeirsson, Christoph Alexander Bauer, and Stefan Grimme*

Mulliken Center for Theoretical Chemistry, Institut für Physikalische und Theoretische Chemie der Rheinischen Friedrich-Wilhelms-Universität Bonn, Beringstr. 4, D-53115, Bonn, Germany

E-mail: grimme@thch.uni-bonn.de

Phone: +49 (0)228/73-2351. Fax: +49 (0)228/73-9064

*To whom correspondence should be addressed

Contents

1 Exemplary reaction coordinates for decomposition pathways **2**

2 Additional Spectra **7**

2.1 Effect of Simulation Time and IEE Distribution 7

2.2 Additional Calculated Spectra of Organic Molecules 11

2.3 Spectra of Organometallic Molecules with ΔSCC (GFN-xTB) IP Evaluation 16

2.4 Additional Calculated Spectra – Deficiencies of MS(GFN-xTB) 18

2.5 Additional Calculated Spectra – Comparison of Semi-empirical PES 23

3 Computational statistics **27**

1 Exemplary reaction coordinates for decomposition pathways

For the prediction of EI-MS by QCEIMS, the quality of the resulting spectra is reflected in the accuracy of the QC method used to compute the atomic forces. In other words, the PES of the QC method has to closely parallel the 'true' PES. Therefore, we compare potential energy curves obtained with GFN-xTB to its level of reference, hybrid DFT. Three simple exemplary reaction pathways involving single bond ruptures are examined: the loss of an ethyl residue from the hexane cation (see Fig. 1), $AsCl_2^+$ from the lewisite cation (see Fig. 2) and iodine from iodobenzene cation (see Fig. 3). Snapshots along the reaction pathway have been superimposed on the figures. The three chosen pathways correspond to the formation of ions which were observed to have relatively intense peak-signals. Therefore, we consider the potential energy curves to be representative of the MD trajectories.

To compute the reaction pathways, we use a simple and intuitive approach, referred to here as a relaxed potential energy surface scan. Given an optimized reactant configuration (the equilibrium ion structure) and products (a neutral and a charged fragment). We perform a linear interpolation of the system with 30 system images placed between the reactant and product states. In the interpolation, only one degree of freedom is varied in an equidistant stepwise fashion, which corresponds to the dissociation process. Each image is then optimized with the dissociating bond distance constrained and all other degrees of freedom are allowed to relax. The optimization is performed using PBE0-D3(bj)/def2-TZVP with an electronic temperature of 10000 K. The energy of each optimized image (including the reactant and product configurations) is calculated with GFN-xTB (at 5000 K) and refined by PBE0-D3(bj)/def2-QZVP (at 10000 K). This methodology for computation of reaction pathways is known to fail for more complex reactions (*i.e.* reaction coordinates) than the ones presented here. This can be seen by an introduction of discontinuity in the potential energy curve, where relaxation of the remaining degrees of freedom pulls the system away from the minimum energy path.

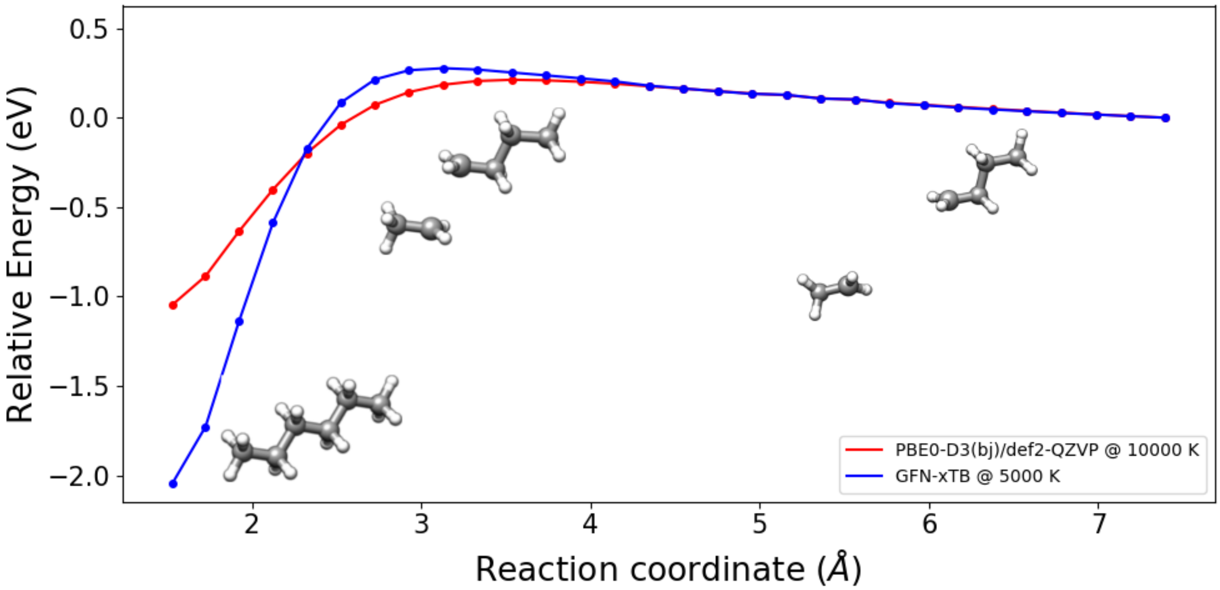

Figure 1: Potential energy curve for the loss of an ethyl residue from the hexane cation. The blue points were calculated using GFN-xTB (5000 K) and the red points by PBE0-D3(bj)/def2-QZVP (10000 K). For clarity, three snapshot along the reaction coordinate have been superimposed on the figure.

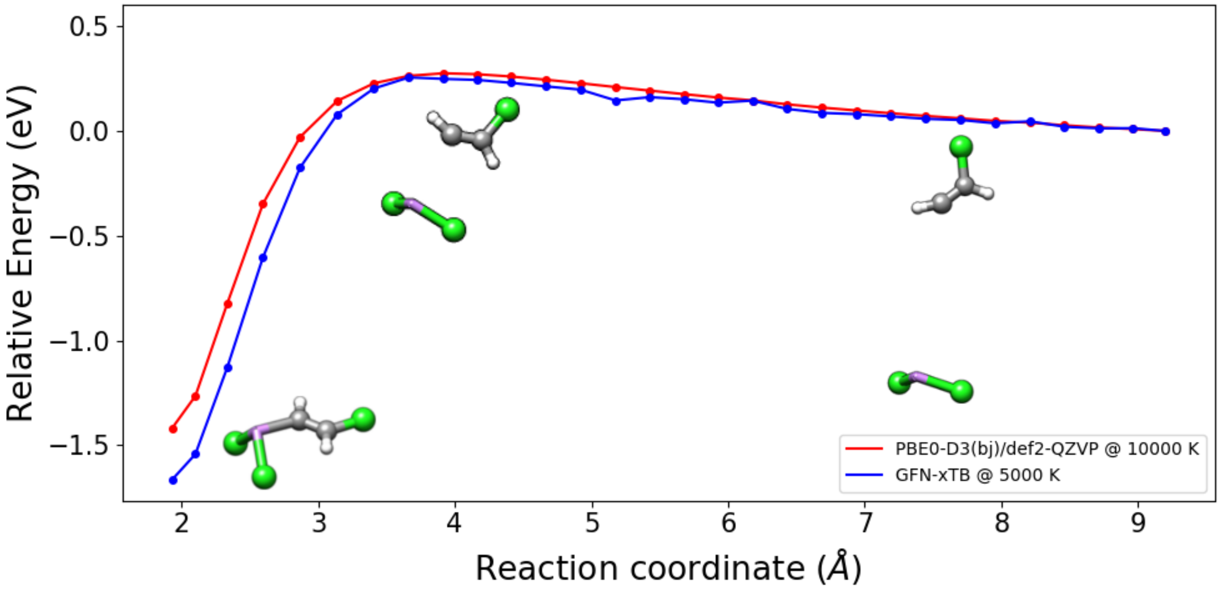

Figure 2: Potential energy curve for the loss of $AsCl_2^+$ from the lewissite cation. The blue points were calculated using GFN-xTB (5000 K) and the red points by PBE0-D3(bj)/def2-QZVP (10000 K). For clarity, three snapshot along the reaction coordinate have been superimposed on the figure.

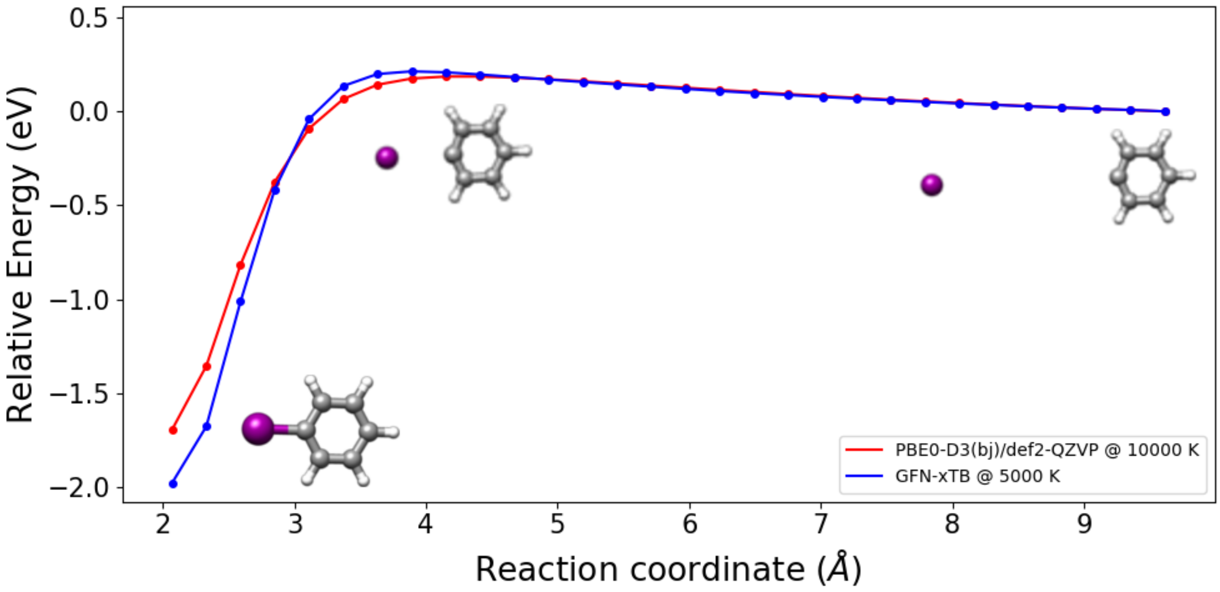

Figure 3: Potential energy curve for the loss of an iodine from the iodobenzen cation. The blue points were calculated using GFN-xTB (5000 K) and the red points by PBE0-D3(bj)/def2-QZVP (10000 K). For clarity, three snapshot along the reaction coordinate have been superimposed on the figure.

The agreement between the shape of the potential energy curves calculated with PBE0-D3(bj)/def2-QZVP and GFN-xTB is excellent, for all three reactions. However, we find the GFN-xTB to predict too strong binding, where the difference can range from roughly 0.25 (lewissite) to 1 eV (hexane).

The three cases shown here are only an initial assessment of GFN-xTB. It is nowhere near complete and a more extensive study is needed *e.g.*, by inclusion of a large number of representative 'real-world' systems and reactions with more complicated reaction coordinates and comparison to high-level *ab-initio* QC calculations and hybrid DFT.

2 Additional Spectra

All additional EI mass spectra are calculated with the same simulation parameters as in the main manuscript, except for the specific modifications, which are investigated in the first subsection.

2.1 Effect of Simulation Time and IEE Distribution

We have investigated the effect of two important simulation parameters: (i) the maximum simulation time parameter, and (ii) the IEE distribution. The former determines one stop criterion in the QCEIMS production runs. It has been set to 10 ps for the results reported in the main manuscript. The latter determines the amount of internal energy deposed in each production run. It is set by default to have its maximum at 0.6 eV per atom. For details, see *Angew. Chem. Int. Ed.*, **2013**, *52*, 6306.

We have scanned these two simulation parameters in the following way : (i) the maximum simulation time is set to 5 ps, 10 ps, and 20 ps, respectively. (ii) The IEE distribution has been set to 0.6 eV per atom (the default value), and 0.3 eV per atom. The results are presented in Figures 4, and 5, respectively. This procedure was performed for the molecules 1-fluorohexane (**2**) and tetramethylsilane (**13**).

The results reveal that the simulation results are perhaps unexpectedly quite robust with respect to the choice of the two parameters. There are, of course, minor differences in the calculated EI-MS of the two compounds, but these are not visible in Figures 4 and 5, but are recorded in the respective output files. Since the purpose of QCEIMS is not to obtain a quantitatively accurate prediction of an EI-MS but rather to obtain a computed spectrum by which a compound may be identified and its unimolecular fragmentation pathways upon electron ionization explored, the finding that the variation of simulation parameters may not

change the results significantly adds to our conclusion that QCEIMS is a stable and reliable program. The systematic exploration of much longer simulation times of 100 ps to a full nanosecond will be the subject of further research, which is beyond the scope of the present study.

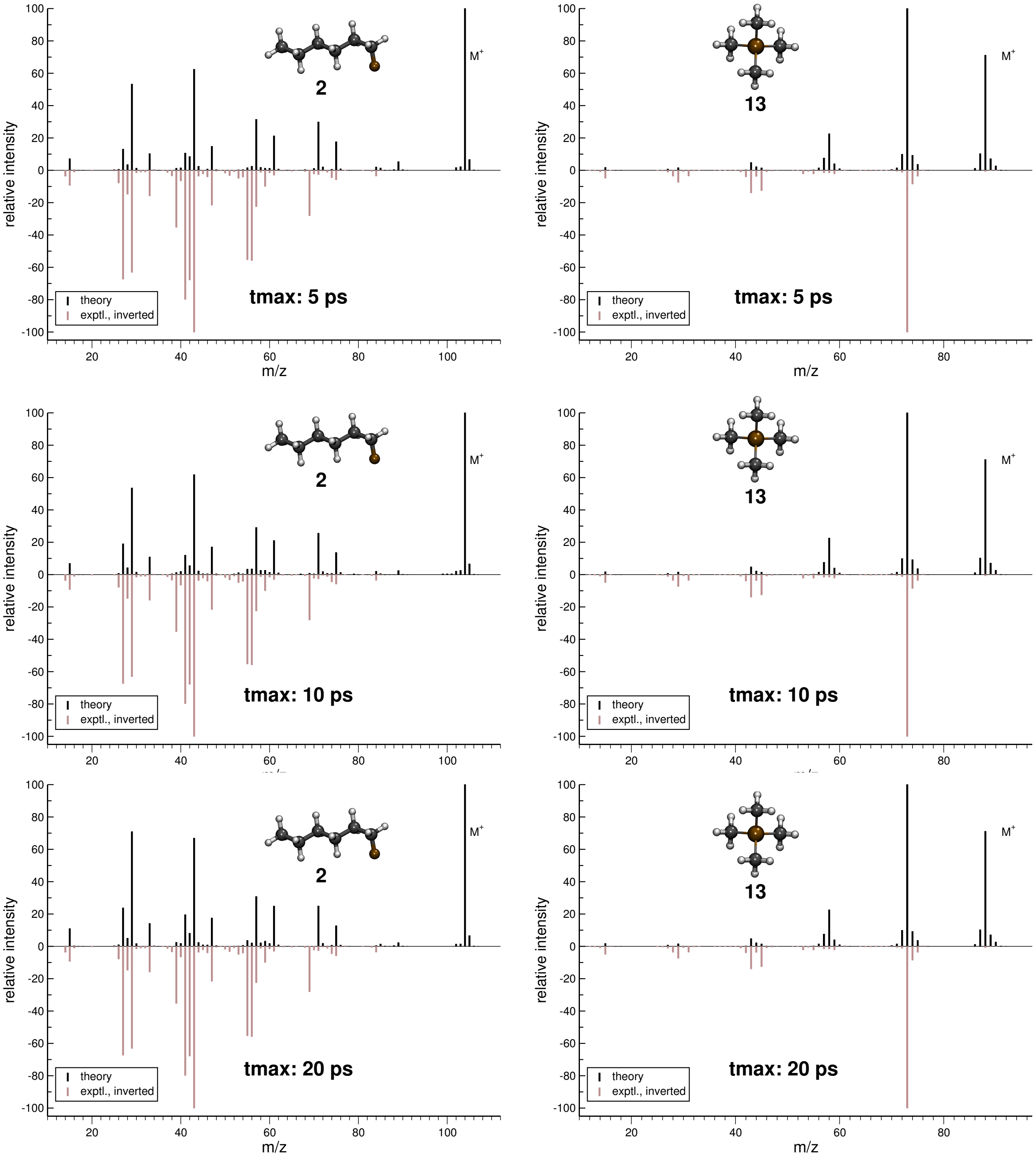

Figure 4: Comparison of computed and experimental EI-MS (GFN-xTB) for 1-F-hexane and tetramethylsilane depending on the maximum simulation time. Maximum of the IEE distribution at 0.6 eV per atom.

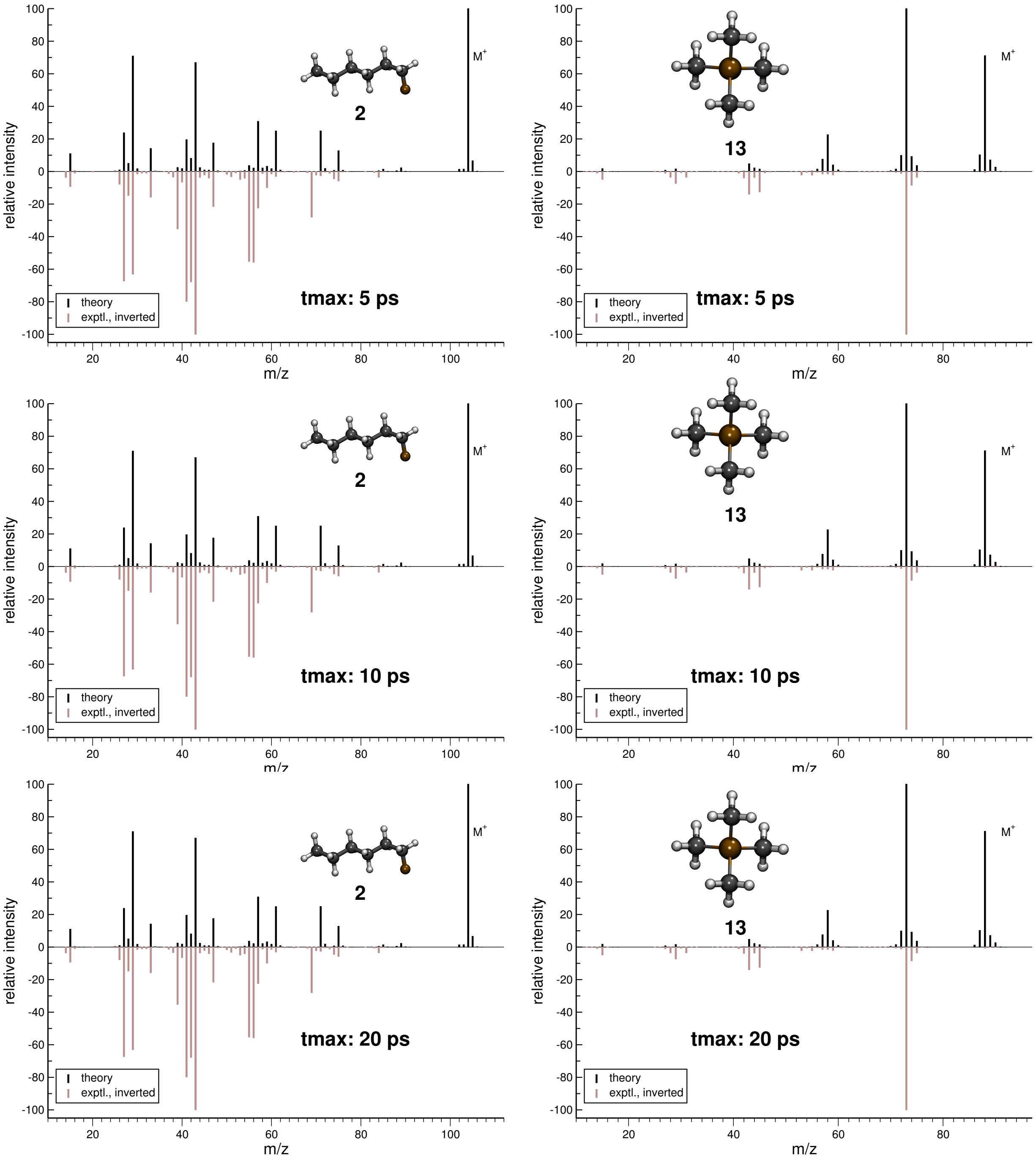

Figure 5: Comparison of computed and experimental EI-MS (GFN-xTB) for 1-F-hexane and tetramethylsilane depending on the maximum simulation time. Maximum of the IEE distribution at 0.3 eV per atom.

2.2 Additional Calculated Spectra of Organic Molecules

We show additional calculated spectra of organic molecules below.

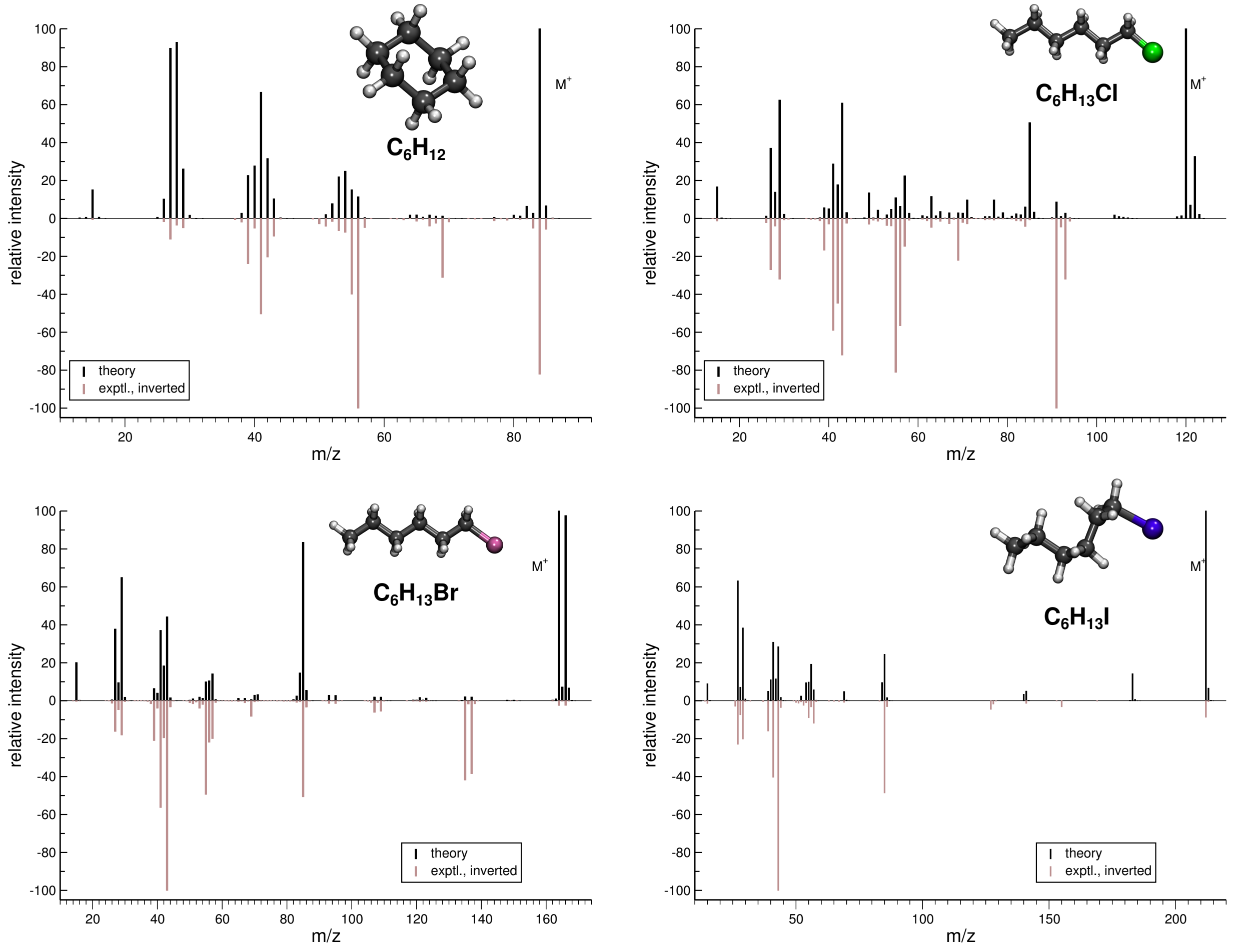

Figure 6: Comparison of computed and experimental EI-MS (GFN-xTB) for simple organic aliphatic and aliphatic halogenide molecules.

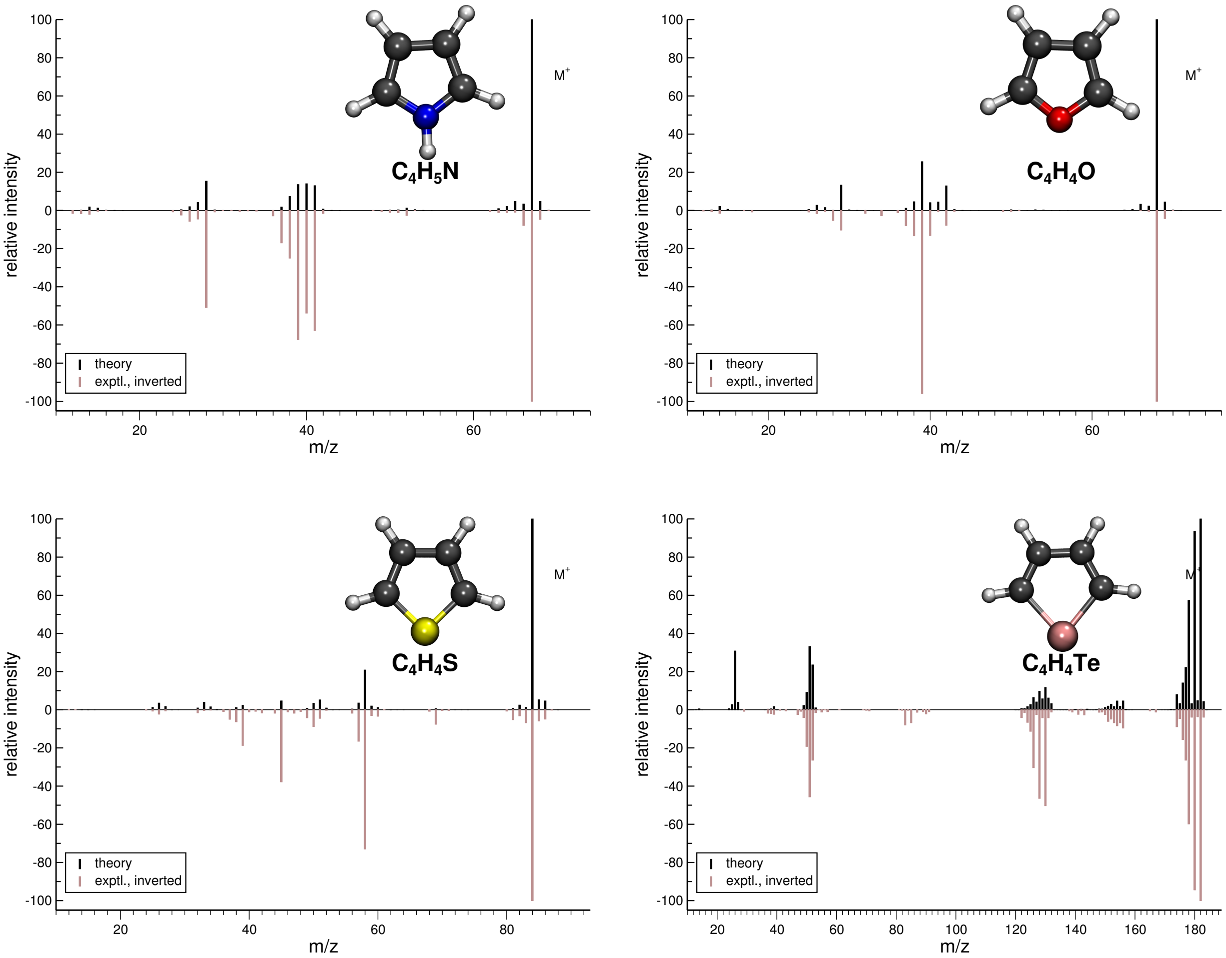

Figure 7: Comparison of computed and experimental EI-MS (GFN-xTB) for aromatic heterocyclic molecules.

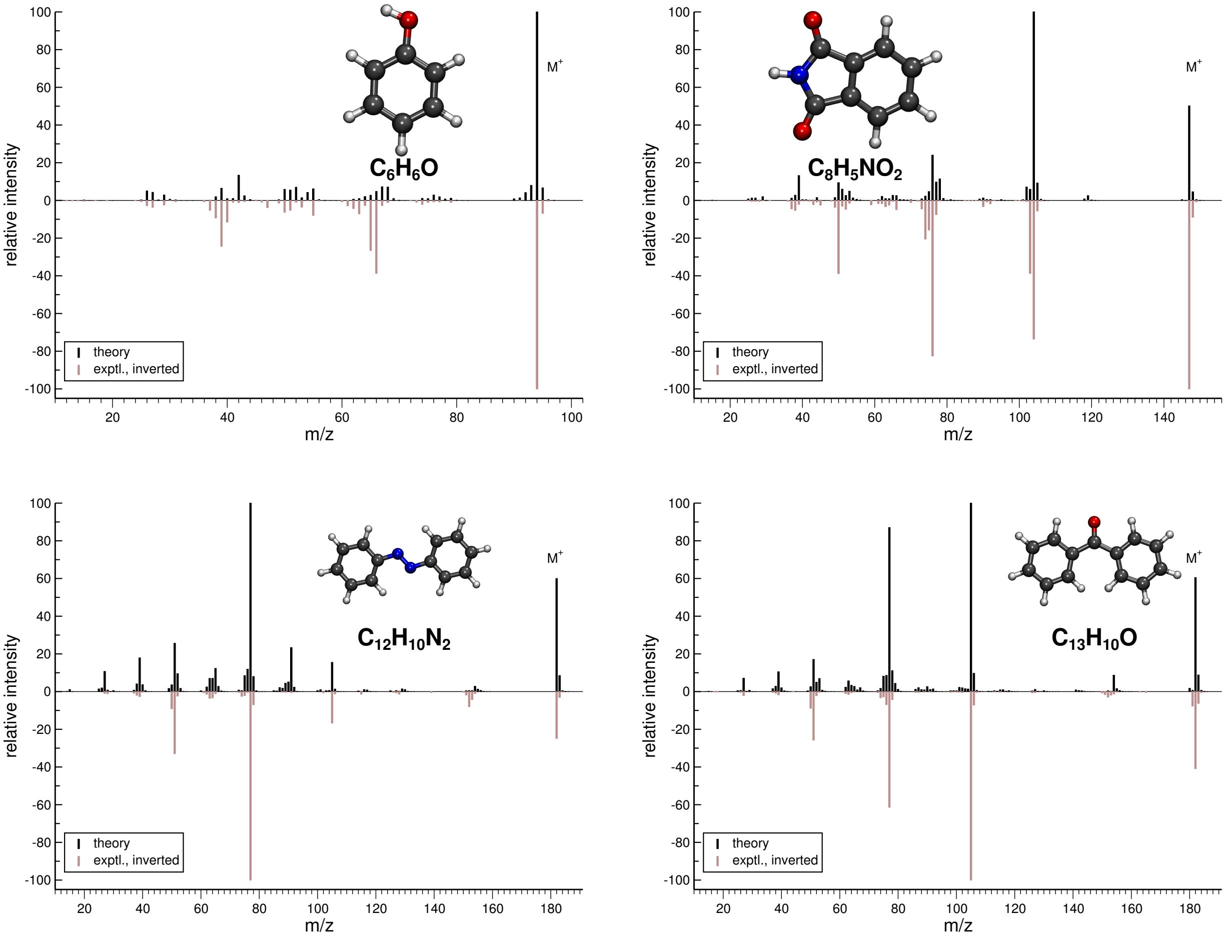

Figure 8: Comparison of computed and experimental EI-MS (GFN-xTB) for aromatic molecules.

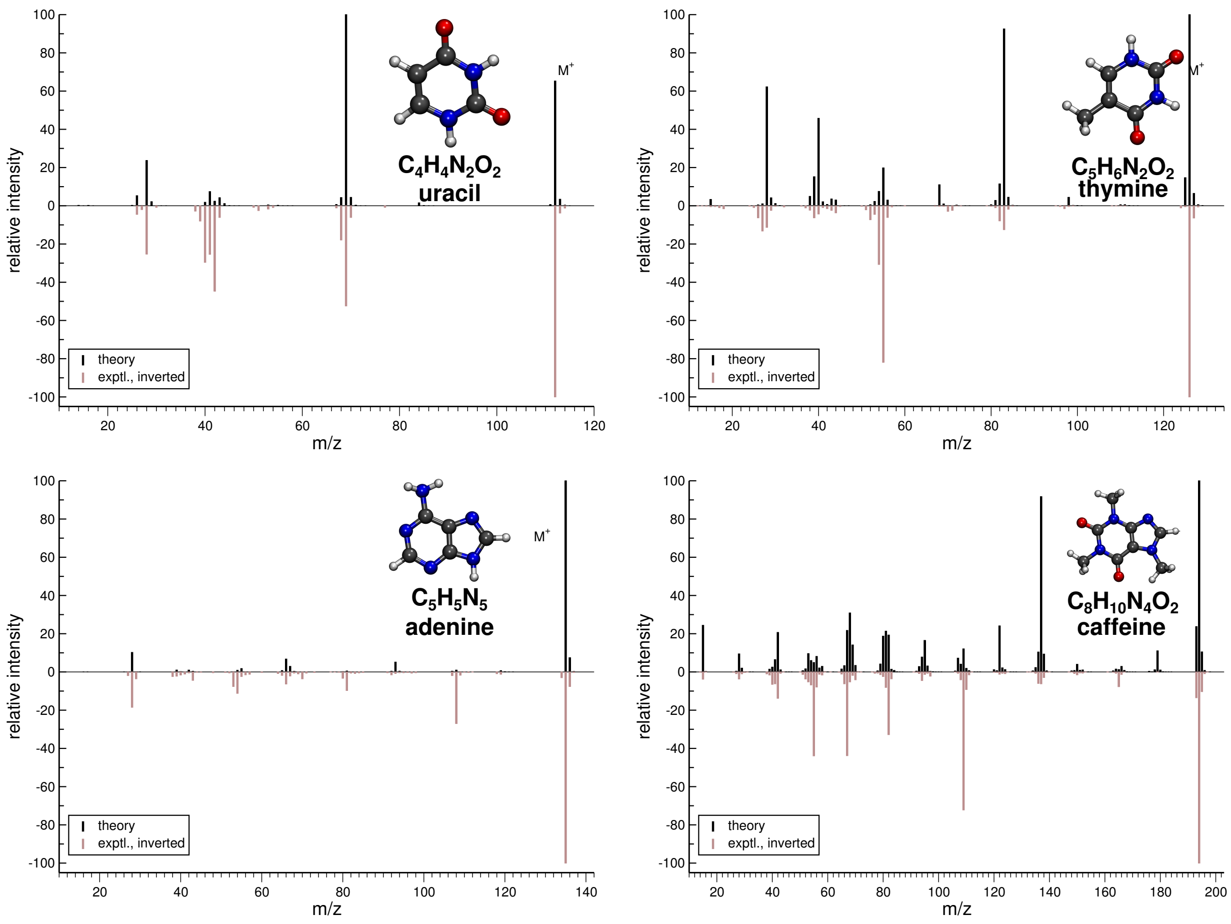

Figure 9: Comparison of computed and experimental EI-MS (GFN-xTB) for pyrimidine and purine derivative molecules.

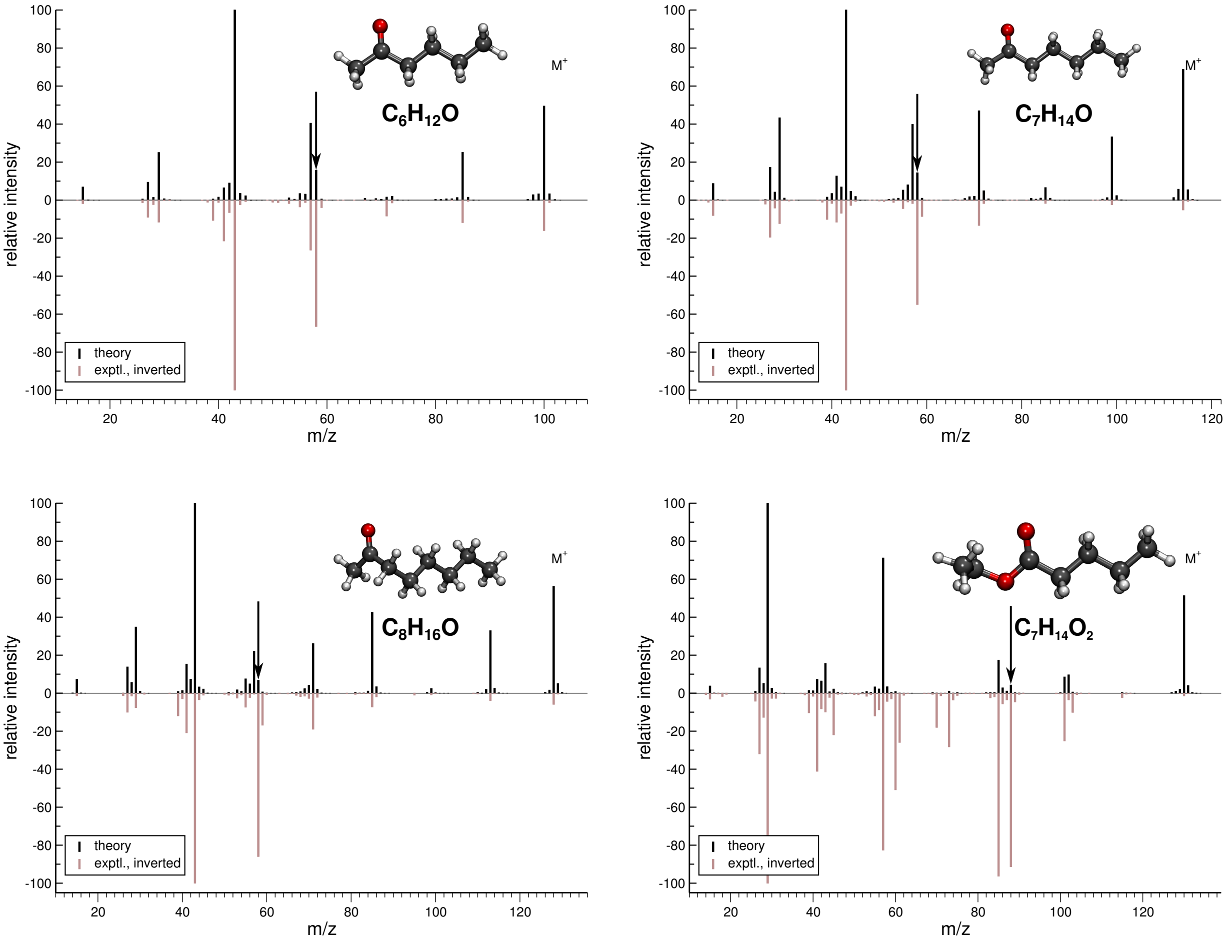

Figure 10: Comparison of computed and experimental EI-MS (GFN-xTB) for molecules that upon ionization undergo the McLafferty rearrangement. The corresponding peaks in the computed EI-MS are marked by the arrows.

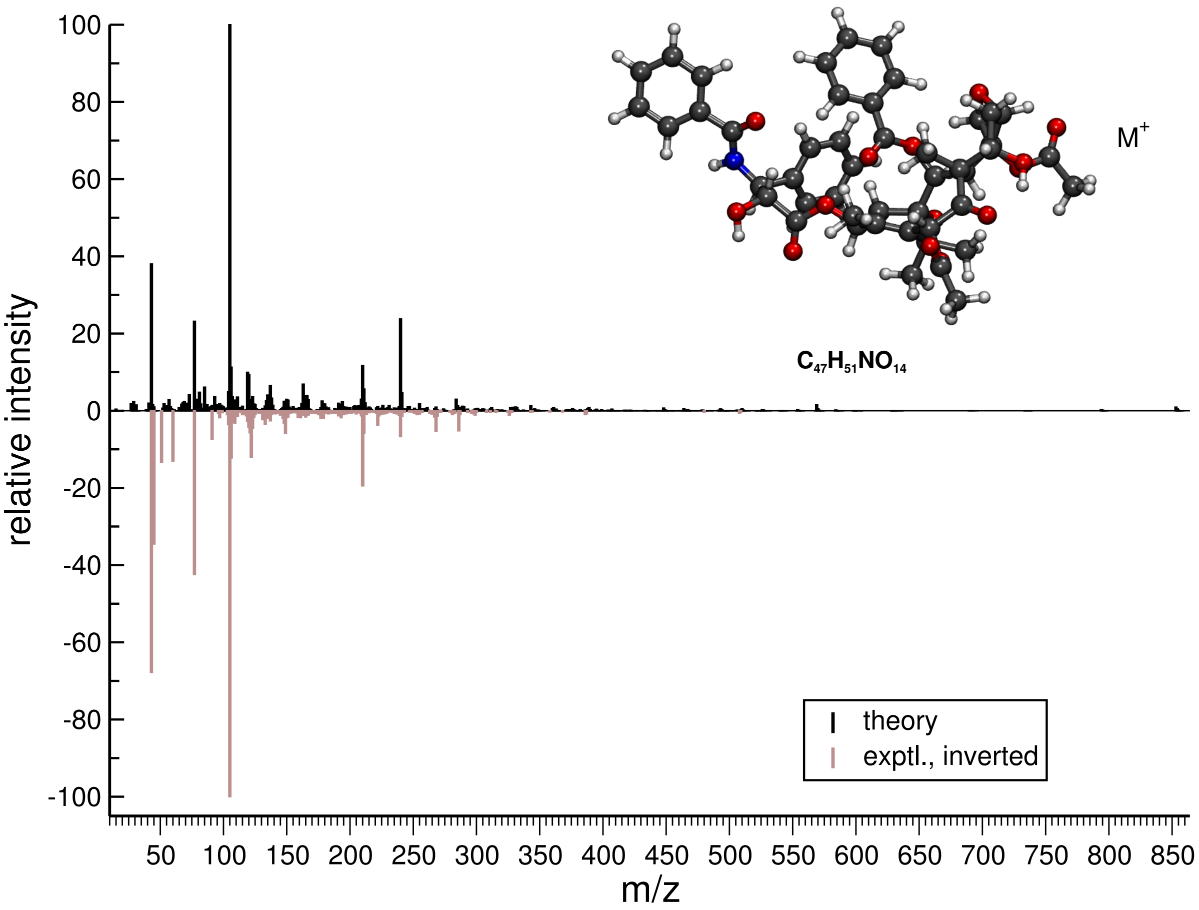

Figure 11: Comparison of computed and experimental EI-MS (GFN-xTB) for taxol.

2.3 Spectra of Organometallic Molecules with ΔSCC (GFN-xTB) IP Evaluation

As seen in Figure 12, the calculated spectrum shows a lot of artifacts that are due to the erroneous evaluation of the fragment ionization potentials, for which no specialized IPEA-xTB parameters exist. In contrast to the spectrum shown in the paper, the naked Fe^{+} is not predicted correctly. For this reason, we recommend that the computation of ionization

potential remain at the Δ SCF (PBE0/SV(P)) level of theory until the parametrization of IPEA-xTB will have been completed.

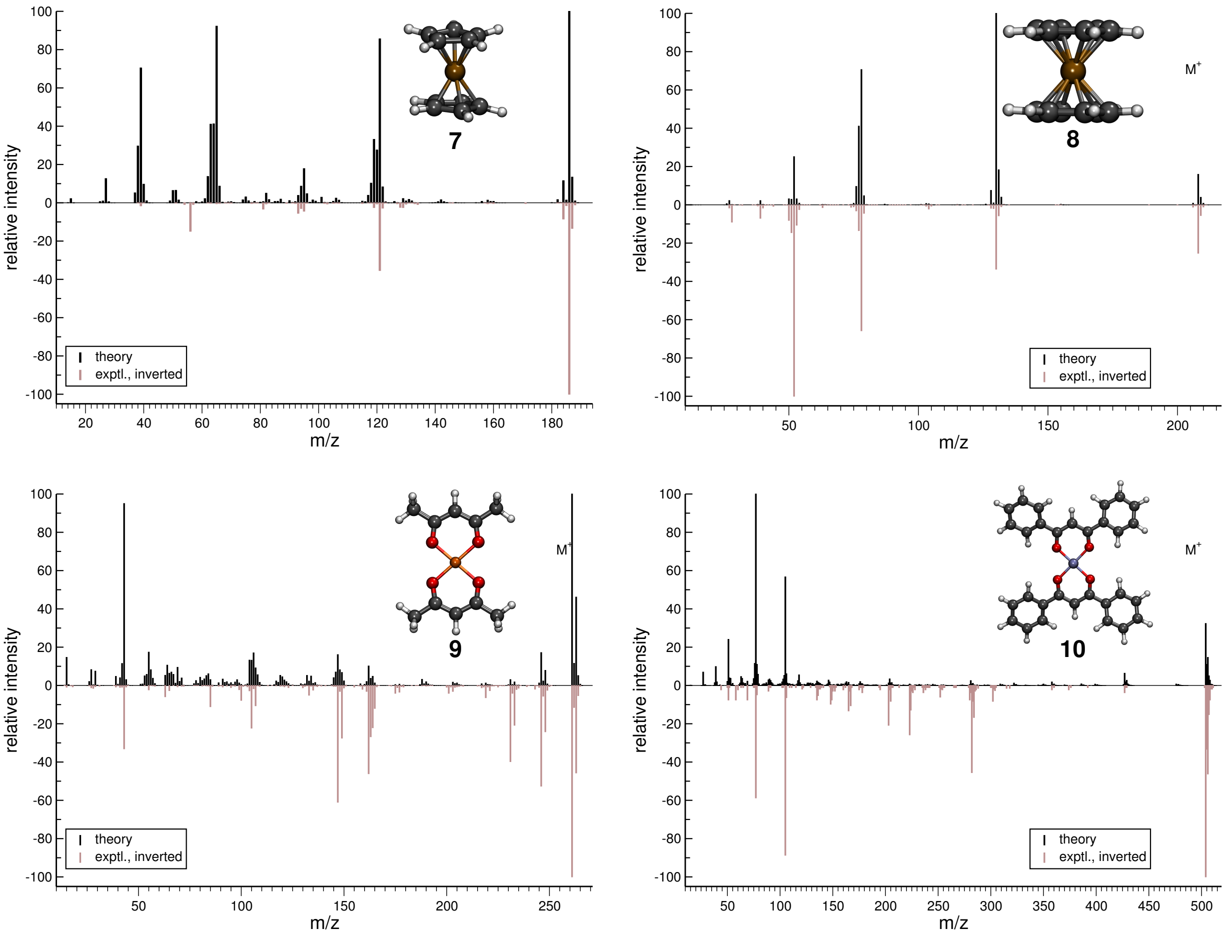

Figure 12: Comparison of computed and experimental EI-MS (GFN-xTB) for the organometallic group using fragment IPs calculated at the Δ SCC (GFN-xTB) level of theory.

The comparisons of computed in experimental EI-MS (GFN-xTB) in Figure 13 reveal that (i) organometallics remain a challenging class of compounds for EI-MS prediction. Yet the problems that are encountered for the systems shown in Figure 13 will be analysed and may thus provide the starting point for the continuous improvement of the QCEIMS method.

This is also valid for the spectra presented in the next subsection.

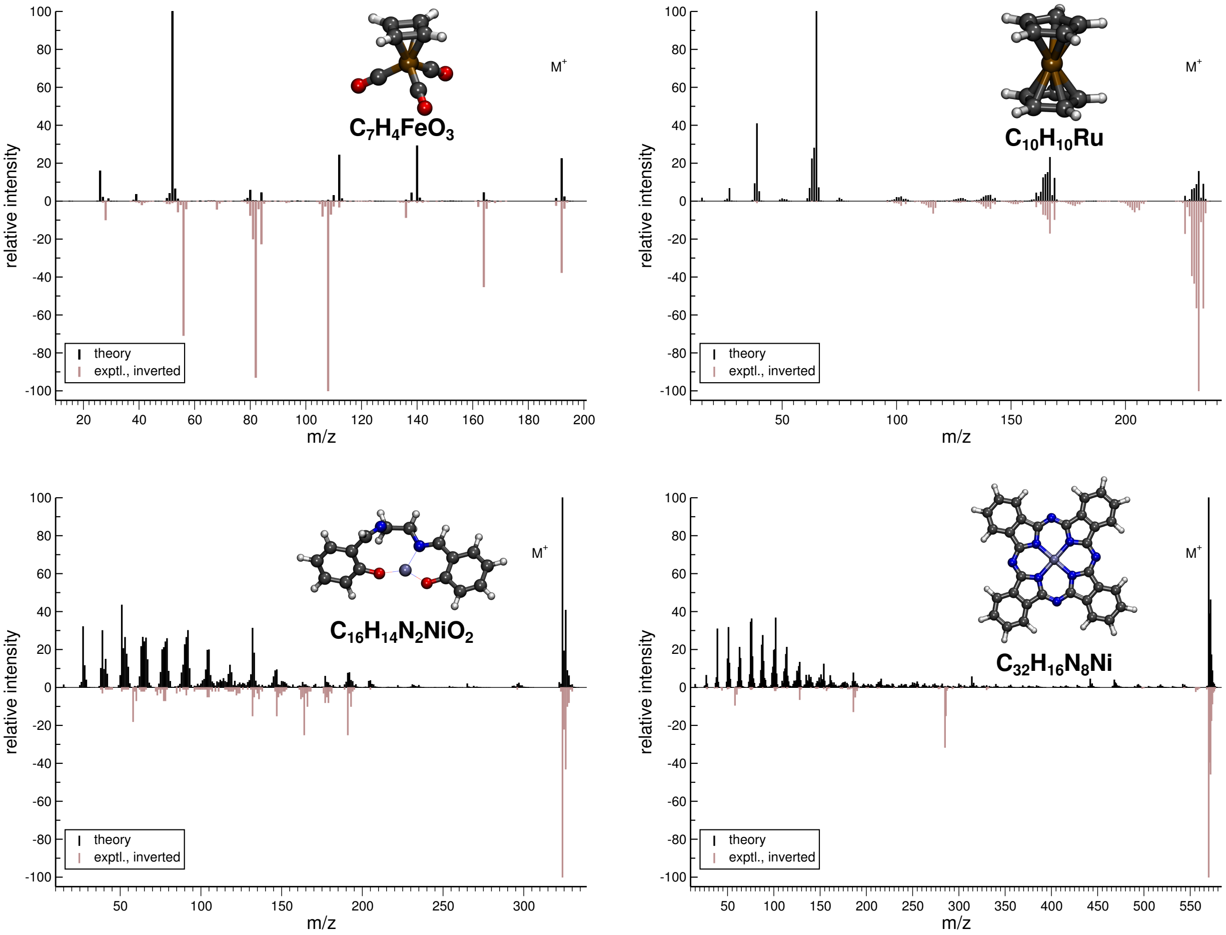

Figure 13: Comparison of computed and experimental EI-MS (GFN-xTB) for additional organometallic molecules using fragment IPs calculated at the Δ SCC (GFN-xTB) level of theory.

2.4 Additional Calculated Spectra – Deficiencies of MS(GFN-xTB)

Here, we present a number of calculated EI-MS, which we do not consider of sufficient quality, and offer preliminary statements on how these failures may be explained. It should be said before all discussion below that GFN-xTB is a semiempirical, cost-efficient QC method,

which cannot be expected to always yield a perfect description of the energetics of the unimolecular fragmentation reaction space.

The apparent failure of EI-MS prediction for several classes of biomolecules by MS(GFN-xTB) (the dipeptides dialanine and cystine, sucrose and tripalmitin, shown in Figure 14) could be considered a distressing finding. However, closer inspection of the simulation results reveals that some of the failures can be explained reasonably. The computed spectrum of dialanine, for instance, consists mostly of the base peak, which is an ion produced in a standard α cleavage channel. It is not unreasonable that the GFN-xTB PES should overrepresent this pathway by perhaps featuring a too low barrier for this reaction. Similar observations are made for sucrose and the triglyceride tripalmitin. For cystine, there are admittedly many artefacts, which, however, disappear when computing the IPs at the Δ SCF (PBE0/SV(P)) level, see Figure 15. That spectrum has been calculated using 200 production runs. The final IP/EA xTB parametrization has not been performed for sulfur yet, and in sensitive cases, we recommend crosschecking the IP evaluation by switching on the Δ SCF (PBE0/SV(P)) level for that part of the simulation.

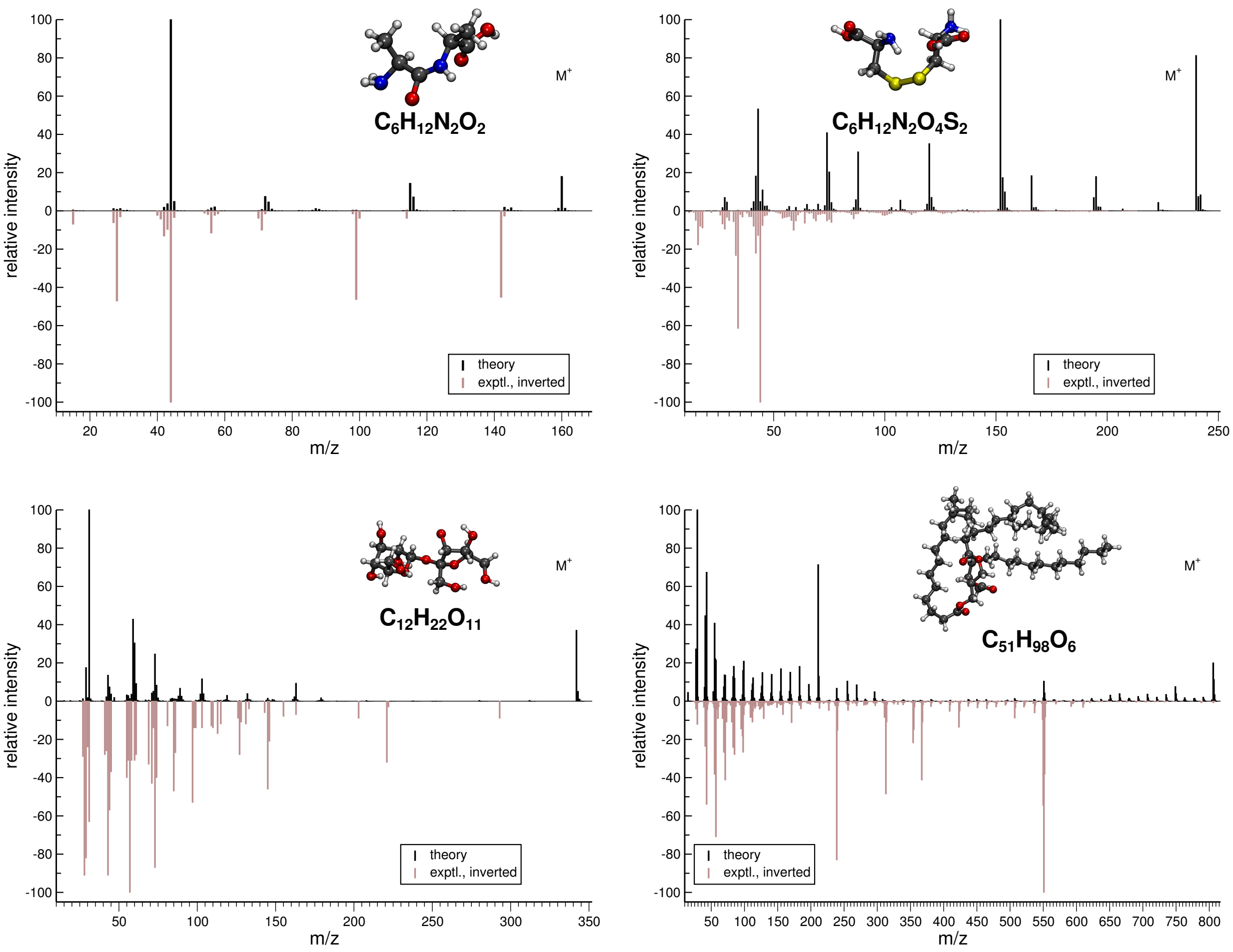

Figure 14: Comparison of computed and experimental EI-MS (GFN-xTB) for different classes of biomolecules.

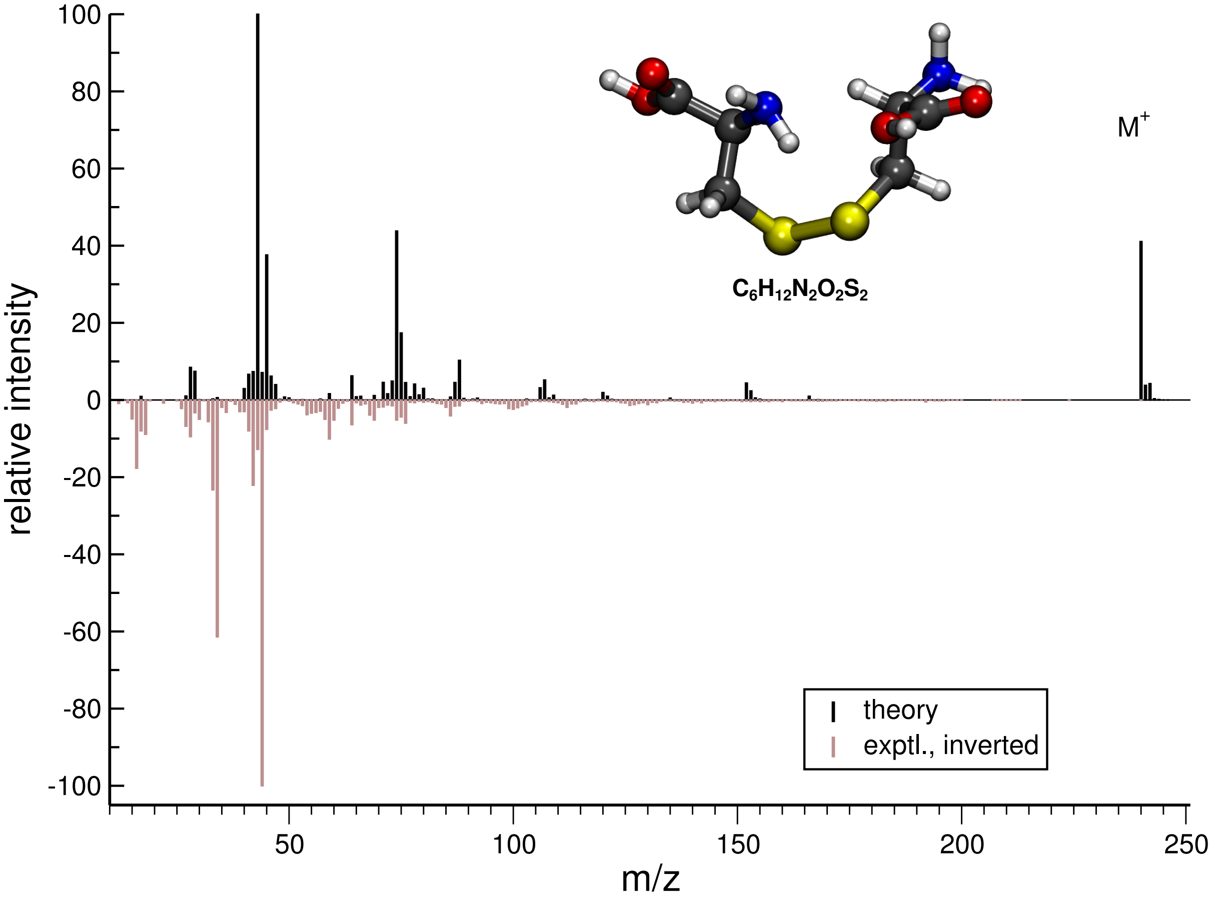

Figure 15: Comparison of computed and experimental EI-MS (GFN-xTB) for cystine with Δ SCF (PBE0/SV(P)) IP evaluation for the fragments.

The computed EI-MS of saframycin A shown in Figure 16 contains a lot of artefacts. Despite the ability of MS(GFN-xTB) to capture some of the main peaks, there are obviously some fragmentation pathways that are artificially overrepresented. Moreover, the internal energy distribution leads in this case to both heavy fragmentation in the production runs as well as survival of the molecular, which is not seen in the experiment. Therefore, the energy distribution may be unbalanced. It will be the topic of further research to investigate why our internal energy distribution model succeeds in many cases but fails in others. Similar

observations are made for tecloftalam (Figure 17).

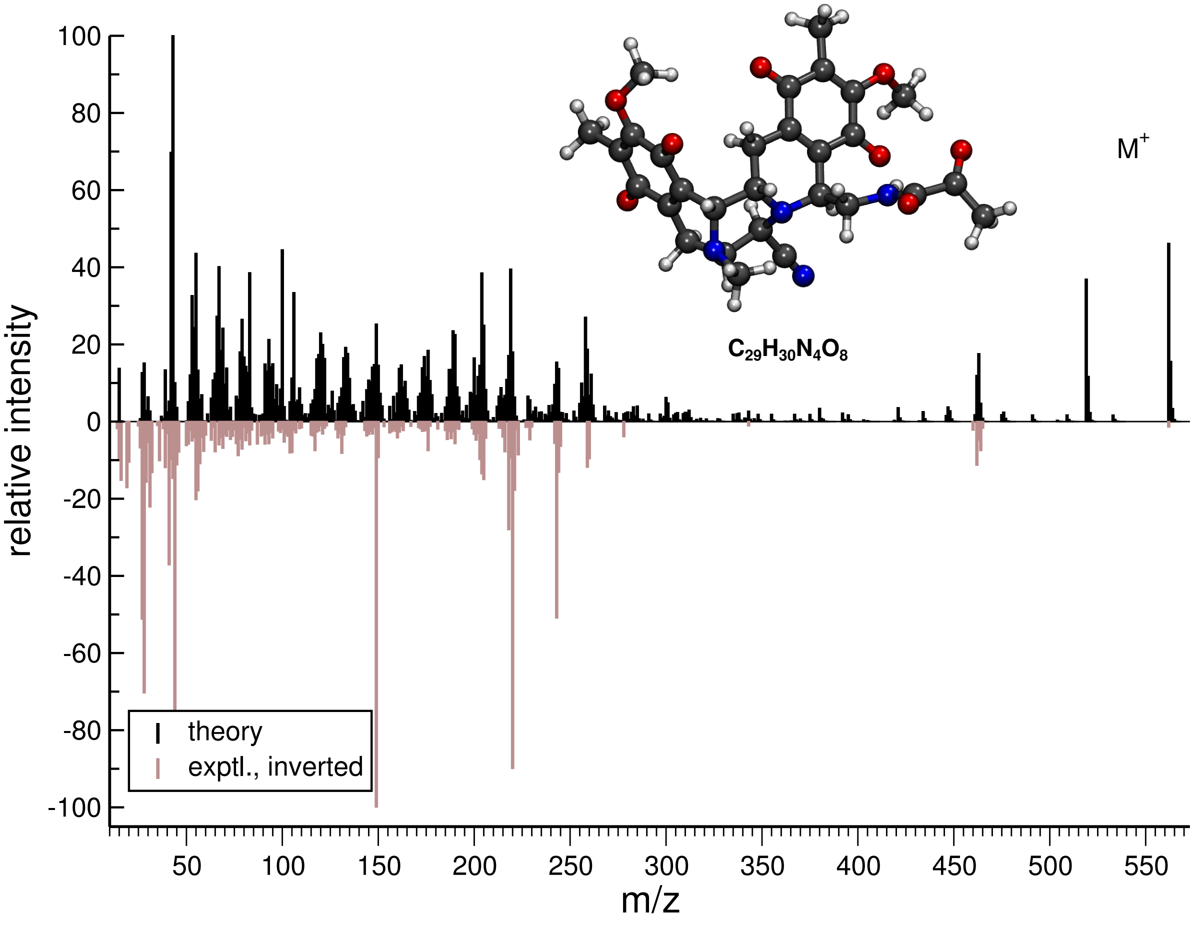

Figure 16: Comparison of computed and experimental EI-MS (GFN-xTB) for saframycin A.

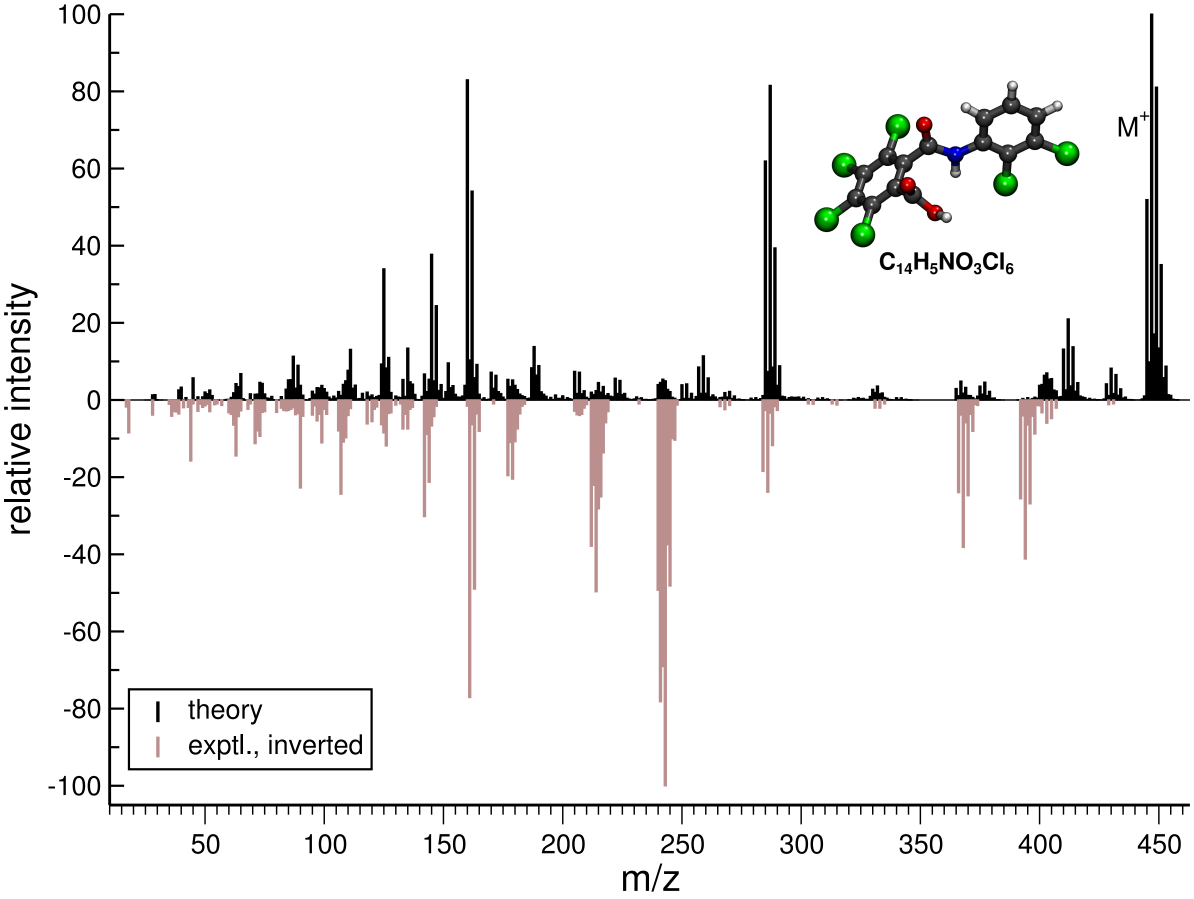

Figure 17: Comparison of computed and experimental EI-MS (GFN-xTB) for tecloftalam.

2.5 Additional Calculated Spectra – Comparison of Semi-empirical PES

In this subsection, we show three illustrative examples of the effect of the semi-empirical quantum chemical PES, which we hold is the largest error source for the computed spectra. The score that is given below is a modified dot-product score. It quantifies the overlap between the computed and experimental spectra. A score of 0 means no overlap, a score of

1,000 means identical spectra.

For the case of methyl sulfonamide, we compare between the DFTB3-D3 and GFN-xTB computed spectra, see Figure 18. For DFTB3-D3, no molecular ion survives the simulation, and the base peak is not identified correctly, indicating that the dissociation energies of the S–N and S–C bonds are not in the right order. The intensity of the peaks in the GFN-xTB computed spectrum is of much higher quality, even if the base peak is misassigned (m/z 15 is the methyl cation, possibly a problem of the IP calculations, as addressed for cystine above). The higher PES quality of GFN-xTB for methyl sulfonamide leads to a much higher score for the comparison between computation and experiment.

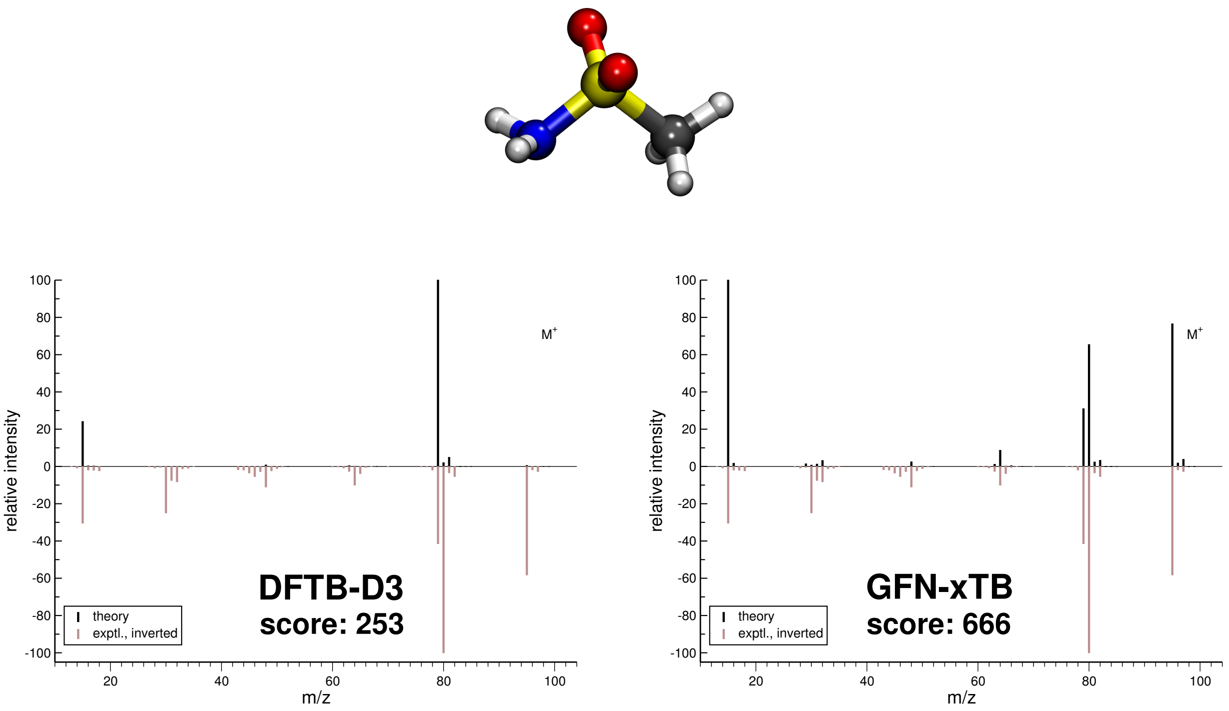

Figure 18: Comparison of computed and experimental EI-MS (DFTB3-D3/GFN-xTB) for methyl sulfonamide.

Figure 19 shows the comparison of DFTB3-D3 and GFN-xTB calculated mass spectra for

2-hexanone. This molecule undergoes a McLafferty rearrangement to yield the ion m/z 58, which is found in 4.6 % of all production runs at the GFN-xTB level of theory, whereas it is not found at all in the DFTB3-D3 production runs. The signal at m/z 58 in the DFTB3-D3 computed spectrum is only due to the isotope peak, which has been added post-simulation. This is another case where the PES quality is the main source of discrepancies between the simulation and the experiment. The McLafferty rearrangement pathway is accessible on the GFN-xTB PES. It appears to be inaccessible on the DFTB3-D3 PES, at least using our standard simulation conditions. Moreover, the base peak, m/z 43, is correctly predicted at the GFN-xTB level of theory whereas the base peak in the DFTB3-D3 computed spectrum is the ion m/z 57. This indicates that the GFN-xTB PES is of a higher quality for 2-hexanone compared to DFTB3-D3, which is also reflected in the spectral matching score difference.

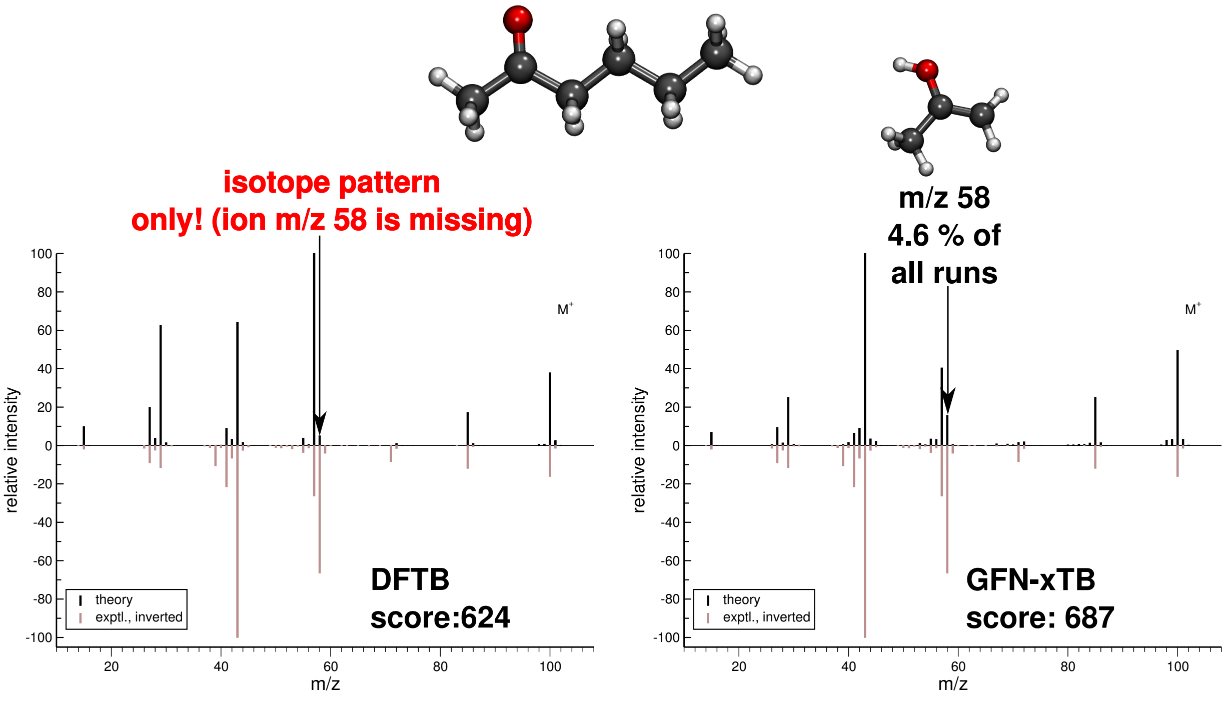

Figure 19: Comparison of computed and experimental EI-MS (DFTB3-D3/GFN-xTB) for 2-hexanone.

Figure 20 reveals how dramatically the quality of the computed spectrum may depend on pair-specific parameters of the GFN-xTB Hamiltonian. In the left spectrum, which is computed using the standard GFN-xTB parametrization (and has been part of the first submission of this manuscript), there are a lot of artifacts, especially the ion m/z57 (Fe-H^+). This artifact is completely removed in the new spectrum (which is now part of the main manuscript), simply by scaling down the Fe-H pair-specific parameter, which can be conveniently done via the parameter file of GFN-xTB read in by the program. The overall quality of the spectrum has thus greatly improved. Future research will be carried out in other cases where the standard parametrization of GFN-xTB, which has provided excellent results, see the spectra in the main part of the manuscript, apparently fails.

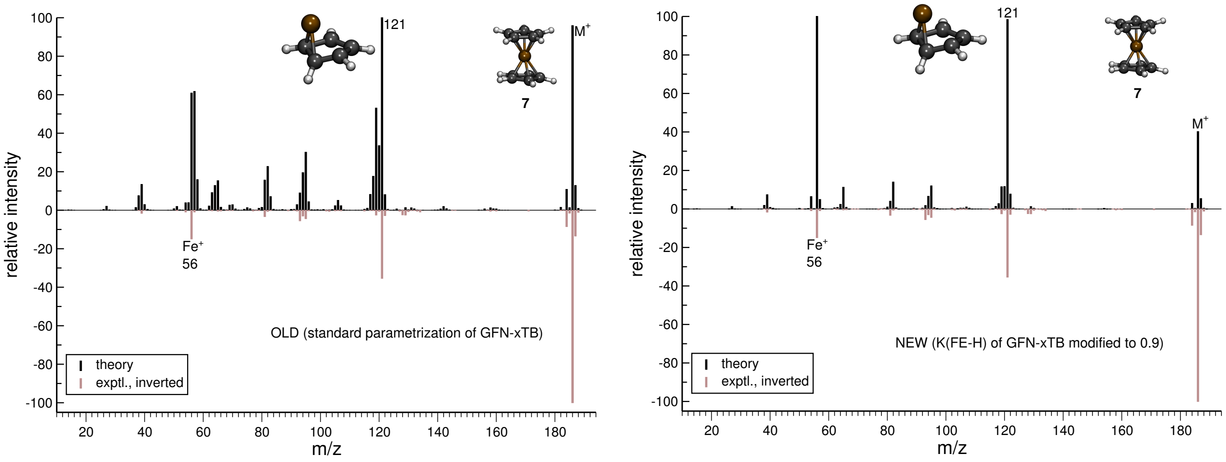

Figure 20: Comparison standard and slightly modified GFN-xTB PES computed and experimental EI-MS (GFN-xTB/IP: PBE0/SV(P)) for ferrocene.

Lastly, we show a comparison between PM6-D2H and GFN-xTB calculated spectra of ferrocene in Figure 21. The discussion here focuses on the ion m/z 105, FeC_4H^+, which is found in traces in the experimental spectrum. As displayed in Figure 21, this ion has a chemically unreasonable structure, which is due to the short-range deficiencies of the PM6-

D2H Hamiltonian (in essence, there is no Pauli repulsion), manifesting itself in the artificially short Fe–C bond length of 1.05 Å. The FeC_4H^+ ion also appears in the GFN-xTB calculated spectrum, although only as the results of one production run. Its structure is much more reasonable with a Fe–C distance of 1.97 Å. We therefore argue that GFN-xTB may produce artifacts, but they are to the best of our knowledge 'reasonable' artifacts, *e.g.*, due to a wrong ordering of reaction channels on the PES. We have not observed any completely unphysical structures of our simulated fragment ions.

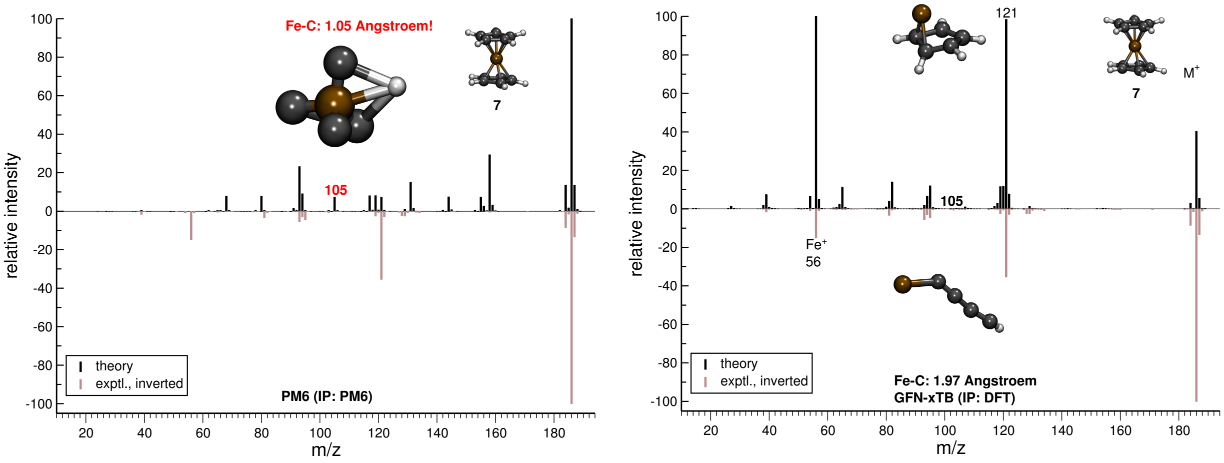

Figure 21: Comparison of computed and experimental EI-MS (PM6-D2H/GFN-xTB) for ferrocene.

3 Computational statistics

The average computational time required for a single point energy/gradient computation (QC call) and the number of unsuccessful production runs is depicted in Figure 3 (in the paper) for each molecule, with the exclusion of nickel(II)bis(diphenyl-acetylacetonate). This average computational time is calculated by the ratio of the total wall-time and the total number of QC calls, over all 1000 production runs. Moreover, for further transparency we

report here (see Table 1) the maximum and average number of QC calls per production run as well as the average and maximum computational time. The data show the large spread of computation times in the production runs depending on the fragmentation events and the stop criteria. The maximum computational time for a production run is often reached when the molecular ion survives while the maximum of QC calls is often related to production runs with many cascading trajectories, which are not necessarily more expensive due to the neutral losses being discounted. Table 1 also reflects that the IP calculation by DFT (as was done for the organometallic systems **7-10** significantly increases the computational times.

Table 1: Average number of energy/gradient computations (QC calls) in a production run along with the standard deviation and the maximum number of QC calls, for the given molecules **1–23**. Furthermore, we show the average (along with standard deviation) and maximum computational time per production run, as well. To obtain an estimate of the total wall time, multiply the average t_{comp} by 1,000 and divide by the number of available cores (which has been, in our case 1,000).

**Molecular index**	**Avg. QC $_{calls}$**	**Max QC $_{calls}$**	**Avg. t_{comp}** [s]	**Max t_{comp}** [s]
**1**	8651.5 ± 6081.5	26319	489.8 ± 395.3	1592
**2**	10840.4 ± 6457.0	29979	670.45 ± 457.0	1633
**3**	7943.6 ± 5870.8	23277	424.48 ± 350.6	1294
**4**	10289.4 ± 5677.3	36008	574.6 ± 366.0	2071
**5**	7302.6 ± 5947.6	36008	99.3 ± 89.8	487
**6**	14312.5 ± 6403.7	44018	857.3 ± 595.6	3649
**7**	9403.9 ± 6423.2	36012	2664.4 ± 2176.4	24299
**8**	7700.9 ± 5336.5	28339	2061.9 ± 1010.7	5463
**9**	11976.1± 6718.1	36012	1627.7 ±939.5	7008
**10**	13622.0 ± 6467.3	33228	18697.2 ± 20969.7	135460
**11**	4536.6 ± 3760.5	22008	179.0 ± 147.7	893
**12**	3949.3 ± 2986.0	20002	183.5 ± 141.1	1031
**13**	12695.6 ± 5896.9	36008	734.0 ± 380.4	2030
**14**	14029.0 ± 5692.1	36008	442.3 ± 215.5	1090
**15**	9765.8 ± 5787.0	20596	538.6 ± 354.4	1278
**16**	10602.4 ± 6193.8	25066	850.6 ± 647.8	2393
**17**	11056.9 ± 5414.6	33222	340.1 ± 199.4	1067
**18**	5011.2 ± 5993.5	21535	85.9 ± 95.2	354
**19**	15218.0 ± 5323.3	29927	604.5 ± 272.2	1094
**20**	13378.5 ± 5770.2	42958	495.0 ± 248.0	1603
**21**	6196.1 ± 5938.1	22008	477.2 ± 481.8	1886
**22**	10457.0 ± 7647	22008	636.85 ± 482.6	1541
**23**	10931.9 ± 7872.8	20002	629.4 ± 490.4	1972
